# Diagnostic tests, drug prescriptions, and follow-up patterns after incident heart failure: A cohort study of 93,000 UK patients

**DOI:** 10.1371/journal.pmed.1002805

**Published:** 2019-05-21

**Authors:** Nathalie Conrad, Andrew Judge, Dexter Canoy, Jenny Tran, Johanna O’Donnell, Milad Nazarzadeh, Gholamreza Salimi-Khorshidi, F. D. Richard Hobbs, John G. Cleland, John J. V. McMurray, Kazem Rahimi

**Affiliations:** 1 The George Institute for Global Health, University of Oxford, Oxford, United Kingdom; 2 Nuffield Department of Orthopaedics, Rheumatology and Musculoskeletal Sciences, Nuffield Orthopaedic Centre, University of Oxford, Oxford, United Kingdom; 3 Bristol National Institute for Health Research Biomedical Research Centre, Musculoskeletal Research Unit, University of Bristol, Southmead Hospital, Bristol, United Kingdom; 4 Medical Research Council Lifecourse Epidemiology Unit, University of Southampton, Southampton General Hospital, Southampton, United Kingdom; 5 National Institute for Health Research Oxford Biomedical Research Centre, University of Oxford, Oxford, United Kingdom; 6 Deep Medicine, Oxford Martin School, University of Oxford, Oxford, United Kingdom; 7 Collaboration Center of Meta-Analysis Research, Torbat Heydariyeh University of Medical Sciences, Torbat Heydariyeh, Iran; 8 Nuffield Department of Primary Care Health Sciences, Radcliffe Observatory Quarter, University of Oxford, Oxford, United Kingdom; 9 Robertson Centre for Biostatistics and Clinical Trials, University of Glasgow and National Heart & Lung Institute, Imperial College London, London, United Kingdom; 10 Institute of Cardiovascular and Medical Sciences, University of Glasgow, Glasgow, United Kingdom; 11 Oxford University Hospitals National Health Service Foundation Trust, Oxford, United Kingdom; Edinburgh University, UNITED KINGDOM

## Abstract

**Background:**

Effective management of heart failure is complex, and ensuring evidence-based practice presents a major challenge to health services worldwide. Over the past decade, the United Kingdom introduced a series of national initiatives to improve evidence-based heart failure management, including a landmark pay-for-performance scheme in primary care and a national audit in secondary care started in 2004 and 2007, respectively. Quality improvement efforts have been evaluated within individual clinical settings, but patterns of care across its continuum, although a critical component of chronic disease management, have not been studied. We have designed this study to investigate patients’ trajectories of care around the time of diagnosis and their variation over time by age, sex, and socioeconomic status.

**Methods and findings:**

For this retrospective population-based study, we used linked primary and secondary health records from a representative sample of the UK population provided by the Clinical Practice Research Datalink (CPRD). We identified 93,074 individuals newly diagnosed with heart failure between 2002 and 2014, with a mean age of 76.7 years and of which 49% were women. We examined five indicators of care: (i) diagnosis care setting (inpatient or outpatient), (ii) posthospitalisation follow-up in primary care, (iii) diagnostic investigations, (iv) prescription of essential drugs, and (v) drug treatment dose. We used Poisson and linear regression models to calculate category-specific risk ratios (RRs) or adjusted differences and 95% confidence intervals (CIs), adjusting for year of diagnosis, age, sex, region, and socioeconomic status. From 2002 to 2014, indicators of care presented diverging trends. Outpatient diagnoses and follow-up after hospital discharge in primary care declined substantially (ranging from 56% in 2002 to 36% in 2014, RR 0.64 [0.62, 0.67] and 20% to 14%, RR 0.73 [0.65, 0.82], respectively). Primary care referral for diagnostic investigations and appropriate initiation of beta blockers and angiotensin-converting–enzyme inhibitors (ACE-Is) or angiotensin receptor blockers (ARBs) both increased significantly (37% versus 82%, RR 2.24 [2.15, 2.34] and 18% versus 63%, RR 3.48 [2.72, 4.43], respectively). Yet, the average daily dose prescribed remained below guideline recommendations (42% for ACE-Is or ARBs, 29% for beta blockers in 2014) and was largely unchanged beyond the first 30 days after diagnosis. Despite increasing rates of treatment initiation, the overall dose prescribed to patients in the 12 months following diagnosis improved little over the period of study (adjusted difference for the combined dose of beta blocker and ACE-I or ARB: +6% [+2%, +10%]). Women and patients aged over 75 years presented significant gaps across all five indicators of care. Our study was limited by the available clinical information, which did not include exact left ventricular ejection fraction values, investigations performed during hospital admissions, or information about follow-up in community heart failure clinics.

**Conclusions:**

Management of heart failure patients in the UK presents important shortcomings that affect screening, continuity of care, and medication titration and disproportionally impact women and older people. National reporting and incentive schemes confined to individual clinical settings have been insufficient to identify these gaps and address patients’ long-term care needs.

## Abstract 2

To investigate trajectories of care for people in the UK with heart failure, Kazem Rahimi & co-workers used linked primary and secondary health records to assess variations over time from diagnosis, and by age, sex and socioeconomic status.

## Author summary

**Why was this study done?:**

- Heart failure is a common, costly, and severe condition—it affects about 2% of the population in high-income countries, with mortality rates comparable to the gravest forms of cancer.
- Effective treatments exist but involve a complex process of investigations, stepwise initiation of medicines, and dose adjustments that in practice is often challenging to follow.
- Over the past decade, the UK introduced separate programmes to evaluate and improve the management of heart failure patients in primary and secondary care. However, patients’ trajectories of care across different healthcare settings have not been studied.

**What did the researchers do and find?:**

- We aimed to investigate the medical care received by heart failure patients in the UK from diagnosis up to a year later.
- We used electronic health records from 93,000 patients who were diagnosed with heart failure between 2002 and 2014. We investigated trajectories of care, including where patients were diagnosed, investigations that were performed, and medications they received, examined changes over time and if results differed according to patients’ age, sex, or socioeconomic status.
- Our results show that the medical care received by heart failure patients presents important gaps. Patients were more likely to be diagnosed in hospital rather than by their general practitioner, received insufficient follow-up after hospitalisation, and dosages prescribed to patients were too low. Moreover, gaps in care were more common in women, older people, and, to some extent, socioeconomically deprived individuals.

**What do these findings mean?:**

- Heart failure care in the UK presents important gaps that pertain to patients’ long-term care needs and are likely to negatively impact patients’ health and quality of life.
- Nationwide quality improvement programmes have failed to identify these gaps, essentially because they evaluate each individual healthcare setting on its own. To further improve patient care, health systems may need to reflect on patients’ journey across the continuum of care.
- Women, older people, and deprived populations are particularly prone to receiving suboptimal care. Research that helps us understand the reasons behind these disparities, or models of care that take each group’s specificities into account, could help.

## Introduction

Over the past 25 years, we have witnessed remarkable developments in clinical interventions that improve symptoms, quality of life, and prognosis in patients with heart failure. However, effective clinical care involves a complex process of investigations, stepwise initiation of medicines, and dose titration that often takes place in different care settings over several months and can be difficult to implement consistently. Although clinical guidelines provide a valuable tool to support physicians in the management of heart failure patients [1–7], ensuring optimal use of evidence-based therapies in routine clinical practice remains a major concern and challenge to health services worldwide [8,9].

In the UK, two major programmes have been introduced to improve physicians’ adherence to evidence-based practices: the ‘Quality and Outcomes Framework’ (QOF) [10], a healthcare reporting and incentive scheme that applies to primary care physicians and was initiated in 2004; and the ‘National Heart Failure Audit’ (NHFA) [11], a large-scale reporting scheme for secondary care launched in 2007. Each scheme individually produces yearly reports on selected heart failure care indicators with very high rates of adherence to clinical guidelines [12,13]. Yet, programmes only consider the clinical setting for which they were designed and have not assessed their broader impact on patient care across the continuum of primary and secondary services, which chronic conditions, such as heart failure, rely on. More generally, similar limitations apply to studies that have investigated heart failure care in Western countries. So far, studies have been largely confined to specific clinical settings, selected cohorts, and limited follow-up information, with no ability to describe and compare patient trajectories following a new diagnosis of heart failure (**S1 Table**). Finally, little is known of variations in care practices by important patient characteristics, such as age or sex.

To address these knowledge gaps, we used a database of linked primary and secondary healthcare records in the UK [14] and performed a longitudinal assessment of outpatient care, covering diagnosis, follow-up, diagnostic investigations, treatment initiation, and dosages in patients with incident heart failure. We further sought to investigate temporal trends and variation by important patient characteristics such as age, sex, and socioeconomic status.

## Methods

### Data source

We used electronic health records from the Clinical Practice Research Datalink (CPRD) from 1 January 1985 to 30 September 2015. The CPRD database contains anonymised patient data from approximately 7% of the current UK population and is broadly representative in terms of age and sex. CPRD is one of the largest databases of longitudinal medical records from primary care in the world and has been validated for epidemiological research for a broad range of conditions [14]. Primary care records include demographic information, consultations, drug prescriptions, diagnostic investigations, and referrals to specialists and were linked to secondary care admission records from Hospital Episodes Statistics (HES), which provides information on hospital admissions and related discharge diagnoses. Scientific approval for this study was given by the CPRD Independent Scientific Advisory Committee (ISAC).

### Study population

Patients were men and women aged 16 and over, with records labelled as ‘acceptable' for research purposes by CPRD quality control [14], approved for CPRD and HES linkage, and registered with their general practice for at least 12 months. Amongst these 4.0 million patients, we identified 93,074 with incident heart failure during the period 1 January 2002 to 31 December 2014. Incident heart failure diagnoses were defined as the first record of heart failure in primary care or hospital admission recorded in any diagnostic position and were identified following previously published methods [15].

### Study outcomes

**Fig 1:**
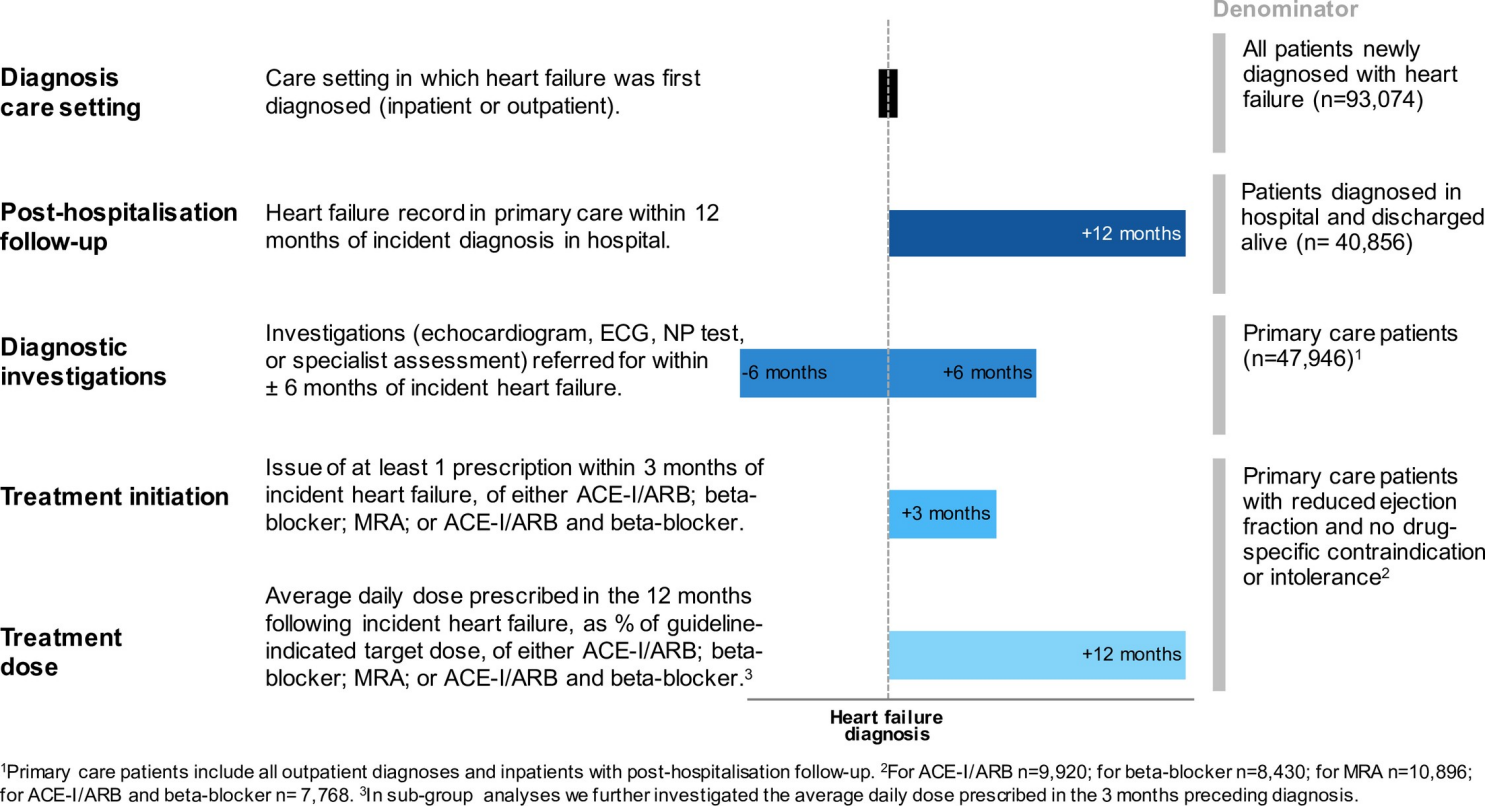
Definitions of essential care indicators. ACE-I, angiotensin-converting–enzyme inhibitor; ARB, angiotensin receptor blocker; ECG, electrocardiogram; MRA, mineralocorticoid receptor antagonist; NP, natriuretic peptide.

We examined five aspects of care across the disease trajectory of patients with heart failure: (i) diagnosis care setting, (ii) posthospitalisation follow-up, (iii) diagnostic investigations, (iv) prescription of essential medicines, and (v) treatment dosages. Data about diagnostic investigations and prescriptions were only available in primary care records, so indicators (iii), (iv), and (v) were restricted to patients whose heart failure was recorded in primary care (*n* = 47,925). Analysis of guideline-recommended drug treatment was further restricted to patients with reduced ejection fraction (*n* = 11,040) with no record of drug-class–specific contraindications or intolerance (**S2**and **S3 Tables**). **Fig 1**provides an overview of study outcome measures, with further details provided below.

The care setting in which heart failure was first diagnosed was categorised as either inpatient or outpatient. Inpatient diagnoses were further categorised based on whether heart failure was listed in primary or secondary diagnostic position. Outpatient diagnoses refer to diagnoses first recorded in primary care with no prior hospitalisation and are likely to reflect both outpatient consultations by specialists and direct diagnoses by general practitioners.

The potential for primary care follow-up after diagnosis in hospital (in short follow-up) was defined as the documentation of heart failure in primary care records within 12 months of diagnosis, with the rationale that documentation of diagnosis is a prerequisite for subsequent disease monitoring and management (**S1 Text**).

Diagnostic investigations included tests recommended as “essential” by the European Society of Cardiology (ESC) guidelines [6]: (i) echocardiogram, (ii) electrocardiogram (ECG), and (iii) plasma natriuretic peptides (B-type natriuretic peptide [BNP] or N-terminal-pro-BNP [NT-pro-BNP]), as well as (iv) cardiology specialist assessment, within ±6 months of incident heart failure diagnosis. Diagnostic tests were considered individually and as a composite of any of the four tests. The list of diagnostic codes used to identify diagnostic tests is presented in **S4 Table**.

Drug treatment patterns were investigated for the three main treatment classes indicated in the management of heart failure with reduced ejection fraction by the ESC guidelines [6]: (i) angiotensin-converting-enzyme inhibitors (ACE-Is) or angiotensin receptor blockers (ARBs), (ii) beta blockers, and (iii) mineralocorticoid receptor antagonists (MRAs).

For each of the three drug classes, we report treatment initiation as the proportion of eligible patients who received at least one prescription in the first three months following their heart failure diagnosis. We defined a composite treatment initiation indicator as prescriptions for the two treatment classes indicated in all patients with heart failure with reduced ejection fraction, which are ACE-Is or ARBs and beta blockers.

We further calculated the average daily dose prescribed to eligible patients over all days alive and registered with their general practice in the first 12 months following incident heart failure (**S2 Text**). We present average daily doses as a fraction of the drug-specific guideline-recommended dose. Daily doses were calculated for all eligible patients, irrespective of receipt of treatment, as an estimate of population level of treatment. The guideline-recommended doses were defined as the minimal target dose recommended by the latest ESC guidelines available during the study period (**S5 Table**). Average daily dose was analysed as a continuous variable, as a binary variable (<50% or ≥50% of recommended dose), as well as categorised into 0%, 1%–24%, 25%–49%, 50%–74%, and ≥75% of the recommended dose. Because drugs used for treatment of heart failure are also used for other indications, we further investigated average daily dose prescribed in the 3 months preceding diagnosis.

### Patient characteristics

We extracted the most recent measurement of baseline characteristics within two years preceding incident heart failure diagnosis including systolic and diastolic blood pressure, smoking status, and body-mass index (BMI). BMI was categorised as underweight (<18.5 kg/m^2^), normal (18.5–24.9 kg/m^2^), overweight (25–29.9 kg/m^2^), and obese (≥30 kg/m^2^).

We further extracted information on comorbidities and socioeconomic status. To describe comorbidities, we selected 17 common chronic conditions (anaemia, asthma, atrial fibrillation, cancer, chronic kidney disease, chronic obstructive pulmonary disease, dementia, depression, diabetes, dyslipidaemia, hypertension, ischaemic heart disease, obesity, osteoarthritis, peripheral arterial disease, stroke, and thyroid disease). Diagnosis code lists for the extraction of each condition were adapted from the CALIBER code repository [16]. To describe socioeconomic status, we used patients’ Index of Multiple Deprivation (IMD) 2015 quintile [17], a composite measure of relative deprivation at a small area level, ranked in ascending order of deprivation score and grouped in equal fifths.

### Statistical analyses

For binary or categorical outcome variables, we computed age, sex, socioeconomic, and year-specific risks as the proportion of eligible patients who received care within a defined time-frame of incident heart failure diagnosis. To assist readability, we further refer to risks, proportions, and rates interchangeably. For continuous outcome variables, we computed means and standard deviations.

To examine changes over time and by subgroups, we used Poisson or linear regression models with robust error variance and report risk ratios (RRs) or adjusted mean differences alongside corresponding 95% confidence intervals (CIs). All models were adjusted for year of diagnosis, age (categorised as <45, 45–54, 55–64, 65–74, and ≥75 years), sex, region, and socioeconomic status. As a measure of temporal trend, we further report *p*-values modelled by including year as a continuous variable. Selected graphical representations were smoothed using local polynomial regression and labelled as such in the figure caption [18,19].

We performed several sensitivity analyses to assess the robustness of our results towards differences in follow-up duration, 30-day mortality, or prevalence of comorbidities over time and by subgroups (**S3 Text**).

Study findings are reported in accordance with the REporting of studies Conducted using Observational Routinely collected health Data (RECORD) recommendations (**S4 Text**) [20]. Analyses were prospectively specified in the study protocol (**S5 Text**), and all statistical analyses were performed in R, version 3.4.2 (R Foundation for Statistical Computing, Vienna, Austria).

## Results

A total of 93,074 patients newly diagnosed with heart failure between 2002 and 2014 were included in the study. Patient characteristics stratified by sex, socioeconomic status, and time period categories have been previously published [15]. Patient characteristics varied depending on where they were first diagnosed; those diagnosed in hospital were older, had more comorbidities, and were more likely to be women (**Table 1**). Patients with a record of reduced ejection fraction were more likely to be younger, be male, and present with fewer comorbidities than those for whom ejection fraction was preserved or unspecified (**S6 Table**).

**Table 1:** Baseline characteristics of patients with incident HF by diagnosis care setting.

Characteristic	All Patients (*n* = 93,074)	Diagnosis Care Setting		
		Inpatient (HF Primary Cause) (*n* = 11,578, 12%)	Inpatient (HF Secondary Cause) (*n* = 40,789, 44%)	Outpatient (Specialist or Primary Care) (*n* = 40,707, 44%)
Age [years], mean (SD)	76.7 (12.6)	78.1 (12.6)	77.6 (12.4)	75.4 (12.7)
Women, no. (%)	45,647 (49%)	6,054 (52%)	20,844 (51%)	18,749 (46%)
Ethnicity, no. (%)				
White	56,011 (88%)	6,974 (88%)	25,259 (88%)	23,778 (87%)
Missing	29,122 (31%)	3,609 (31%)	12,132 (30%)	13,381 (33%)
Socioeconomic status, no. (%)				
1 (least deprived)	18,371 (20%)	2,182 (19%)	7,889 (19%)	8,300 (20%)
2	20,073 (22%)	2,492 (21%)	8,600 (21%)	8,981 (22%)
3	20,052 (22%)	2,479 (21%)	8,697 (21%)	8,876 (22%)
4	18,308 (20%)	2,307 (20%)	8,122 (20%)	7,879 (19%)
5 (most deprived)	16,270 (17%)	2,118 (18%)	7,481 (18%)	6,671 (16%)
Systolic blood pressure				
Mean (SD) [mmHg]	133 (21)	133 (21)	132 (20)	133 (201)
Missing, no. (%)	5,195 (6%)	654 (6%)	2,656 (7%)	1,885 (5%)
Diastolic blood pressure				
Mean (SD) [mmHg]	74 (12)	74 (12)	74 (11)	75 (12)
Missing, no. (%)	5,195 (6%)	654 (6%)	2,656 (7%)	1,885 (5%)
BMI category, no. (%)				
Underweight	2,193 (4%)	284 (4%)	1,107 (5%)	802 (3%)
Normal	17,381 (31%)	2,164 (33%)	7,657 (32%)	7,560 (30%)
Overweight	18,786 (34%)	2,067 (31%)	7,818 (33%)	8,901 (35%)
Obese	17,644 (31%)	2,139 (32%)	7,162 (30%)	8,343 (33%)
Missing	37,070 (40%)	4,924 (43%)	17,045 (42%)	15,101 (37%)
Smoking, no. (%)				
No	29,551 (41%)	3,787 (44%)	12,647 (40%)	13,117 (41%)
Ex	32,572 (45%)	3,683 (43%)	14,299 (46%)	14,590 (46%)
Yes	9,596 (13%)	1,082 (13%)	4,360 (14%)	4,154 (13%)
Missing	21,355 (23%)	3,026 (26%)	9,483 (23%)	8,846 (22%)
Comorbidities				
Atrial fibrillation, no. (%)	36,950 (40%)	5,199 (45%)	17,325 (42%)	14,426 (35%)
Chronic kidney disease, no. (%)	22,762 (24%)	3,233 (28%)	11,336 (28%)	8,193 (20%)
Chronic obstructive pulmonary disease, no. (%)	17,896 (19%)	2,113 (18%)	9,276 (23%)	6,507 (16%)
Diabetes, no. (%)	20,531 (22%)	2,952 (25%)	9,412 (23%)	8,167 (20%)
Dyslipidaemia, no. (%)	25,958 (28%)	3,281 (28%)	12,524 (31%)	10,153 (25%)
Hypertension, no. (%)	62,419 (67%)	8,226 (71%)	28,776 (71%)	25,417 (62%)
Ischaemic heart disease, no. (%)	45,584 (49%)	5,804 (50%)	22,247 (55%)	17,533 (43%)
Osteoarthritis, no. (%)	40,176 (43%)	5,029 (43%)	18,227 (45%)	16,920 (42%)
3 or more comorbidities, no. (%)	73,610 (79%)	9,567 (83%)	34,904 (86%)	29,139 (72%)

Number and percentage of records with missing data are displayed for variables with missing entries. Category percentages refer to complete cases. Socioeconomic status refers to IMD 2015 quintile, with 1 referring to the most affluent and 5 to the most deprived quintile. Number of comorbidities refers to any of the 17 conditions investigated (see Methods). **Abbreviations**: BMI, body-mass index; HF, heart failure; IMD, Index of Multiple Deprivation.

### Temporal trends of essential care indicators

#### Setting of heart failure diagnosis

Between 2002 and 2014, a diagnosis of heart failure was first recorded during a hospitalisation in 56% of cases (12% as a primary diagnosis and 44% as a secondary diagnosis) and in an outpatient setting in 44% (Table 2). Amongst secondary inpatient diagnoses of heart failure, about a third of primary causes for admission were cardiac and a fifth were respiratory disease.

**Table 2:** Temporal trends in essential care indicators following incident HF by year of diagnosis.

	Denominator Cohort (*n*)	2002–2014	2002	2014		*p* for Trend
**Diagnosis Care Setting**	All patients with HF (93,074)	***n* (%)**	***n* (%)**	***n* (%)**	**RR [95% CI]**	
Inpatient (HF primary cause)		11,578 (12%)	912 (12%)	740 (12%)	0.94 [0.86, 1.03]	0.02
Inpatient (HF secondary cause)		40,789 (44%)	2,277 (31%)	3,242 (52%)	1.67 [1.6, 1.74]	<0.001
Outpatient (specialist or primary care)		40,707 (44%)	4,120 (56%)	2,260 (36%)	0.64 [0.62, 0.67]	<0.001
**Posthospitalisation Follow-Up**		***n* (%)**	***n* (%)**	***n* (%)**	**RR [95% CI]**	
All inpatients	Inpatients, discharged alive (40,856)	6,807 (17%)	489 (20%)	459 (14%)	0.73 [0.65, 0.82]	<0.001
Inpatient (HF primary cause)	Primary inpatients, discharged alive (9,195)	2,854 (31%)	228 (32%)	174 (29%)	0.95 [0.80, 1.12]	0.209
Inpatient (HF secondary cause)	Secondary inpatients, discharged alive (31,661)	3,953 (12%)	261 (15%)	285 (11%)	0.73 [0.62, 0.85]	<0.001
**Diagnostic Investigations**	Patients diagnosed or followed up in primary care (47,925)	***n* (%)**	***n* (%)**	***n* (%)**	**RR [95% CI]**	
Echocardiogram		24,649 (51%)	796 (17%)	1,693 (62%)	3.56 [3.36, 3.78]	<0.001
ECG		17,928 (37%)	995 (21%)	1,090 (40%)	1.83 [1.71, 1.96]	<0.001
NP test		4,177 (9%)	NA	616 (23%)	NA	NA
Specialist assessment		14,046 (29%)	563 (12%)	847 (31%)	2.5 [2.27, 2.75]	<0.001
At least 1 diagnostic investigation		33,660 (70%)	1,683 (37%)	2,248 (82%)	2.24 [2.15, 2.34]	<0.001
**Treatment Initiation**	Patients diagnosed or followed up in primary care, reduced ejection fraction (11,040), and no drug-specific contraindication	***n* (%)**	***n* (%)**	***n* (%)**	**RR [95% CI]**	
ACE-I/ARB	9,920	7,748 (78%)	285 (75%)	586 (80%)	1.06 [0.99, 1.13]	<0.001
Beta blocker	8,430	4,661 (55%)	72 (22%)	490 (72%)	3.27 [2.65, 4.03]	<0.001
MRA	10,896	1,999 (18%)	59 (14%)	207 (26%)	1.88 [1.45, 2.44]	<0.001
Beta blocker and ACE-I/ARB	7,768	3,759 (48%)	56 (18%)	403 (63%)	3.48 [2.72, 4.43]	<0.001
**Treatment Dose**	Patients diagnosed or followed up in primary care, reduced ejection fraction (11,040) and no drug-specific contraindication	**mean (SD)**	**mean (SD)**	**mean (SD)**	**Adjusted difference [95% CI]**	
ACE-I/ARB	9,920	48% (45%)	50% (44%)	42% (41%)	−7% [−13%, −2%]	<0.001
Beta blocker	8,430	25% (30%)	11% (22%)	29% (32%)	19% [15%, 22%]	<0.001
MRA	10,896	18% (39%)	16% (36%)	20% (37%)	5% [1, 9%]	<0.001
Beta blocker and ACE-I/ARB	7,768	36% (30%)	30% (26%)	35% (29%)	6% [2%, 10%]	<0.001

RRs or adjusted differences and 95% CIs comparing 2014 to 2002, adjusting for year of diagnosis, age, sex, socioeconomic status, and region. Primary care follow-up refers to the documentation of HF in primary care records during a follow-up consultation within 12 months of an incident diagnosis in hospital. Diagnostic investigations refer to investigations referred for within ±6 months of incident heart failure. Treatment initiation refers to the issue of at least 1 prescription within 3 months of incident heart failure. Treatment dose presents the average daily dose prescribed in the first 12 months following incident heart failure as percent of guideline-recommended target dose. **Abbreviations**: ACE-I, angiotensin-converting–enzyme inhibitor; ARB, angiotensin receptor blocker; CI, confidence interval; ECG, electrocardiogram; HF, heart failure; MRA, mineralocorticoid receptor antagonist; NA, not applicable; NP, natriuretic peptide; RR, risk ratio.

Whilst the proportion of patients diagnosed in hospital with primary heart failure diagnosis remained stable at 12%, there were opposing trends for secondary inpatient diagnoses versus outpatient diagnoses. Rates of outpatient diagnoses showed a 36% relative decline over time (from 56% in 2002 to 36% in 2014, RR 0.64 [0.62, 0.67]) and were offset by a 67% relative increase in secondary inpatient diagnoses (from 31% in 2002 to 52% in 2014, RR 1.67 [1.60, 1.74]) (**Table 2**).

#### Primary care follow-up after hospitalisation

**Fig 2:**
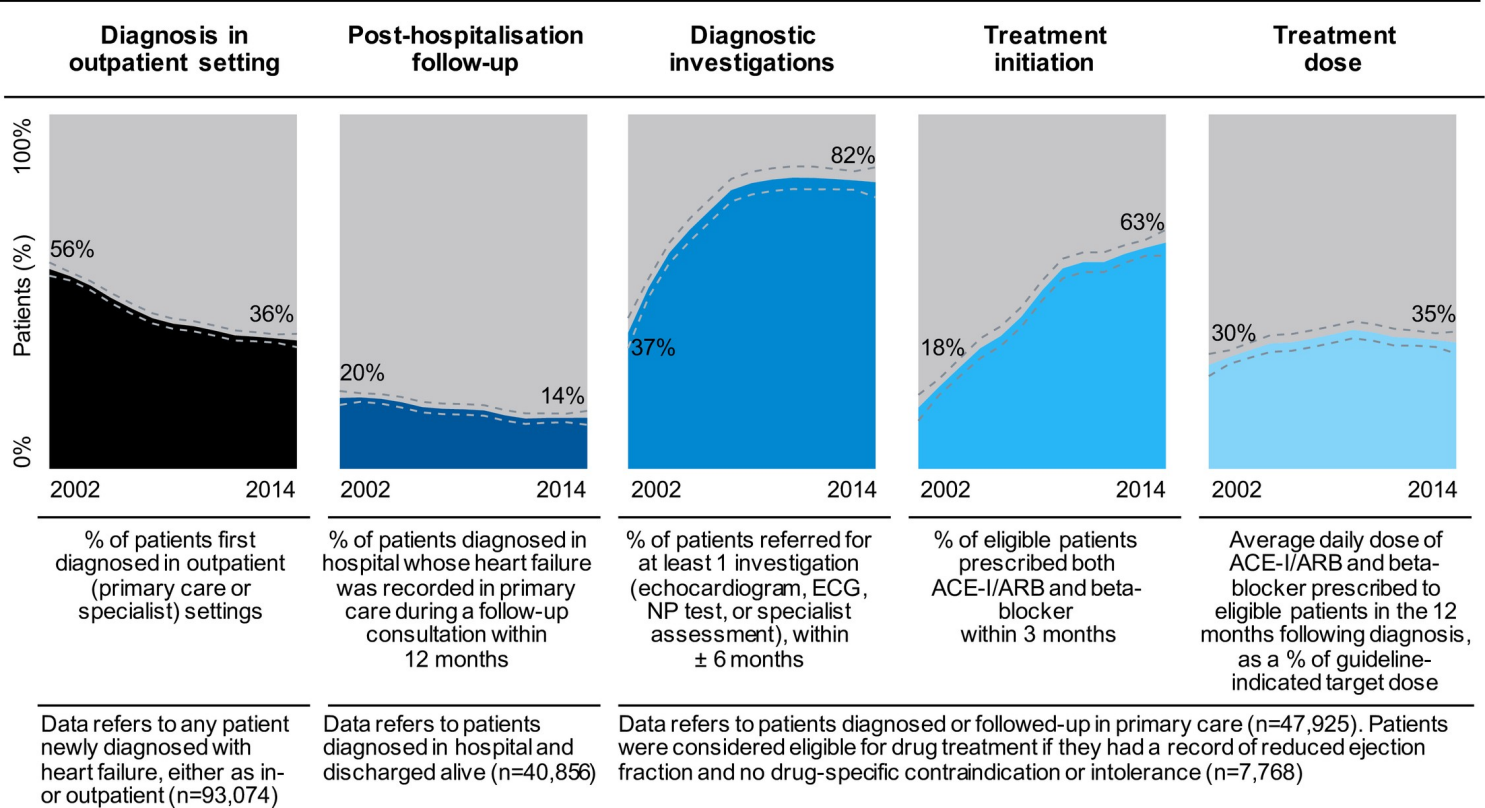
Care delivery indicators following incident heart failure, by year of diagnosis, in CPRD from 2002 to 2014. Results are presented as fitted local polynomial regression over yearly averages and 95% CIs (dashed lines). ACE-I, angiotensin-converting–enzyme inhibitor; ARB, angiotensin receptor blocker; CI, confidence interval; CPRD, Clinical Practice Research Datalink; ECG, electrocardiogram; NP, natriuretic peptide.

Amongst heart failure inpatients who survived the index hospitalisation, only 17% had their heart failure diagnosis recorded by their primary care physician in the subsequent 12 months, with rates declining over time (20% in 2002 versus 14% in 2014; RR 0.73 [0.65, 0.82]) (**Fig 2**). Follow-up rates were higher for patients with a primary discharge diagnosis of heart failure (31%) than secondary diagnosis (12%) (**Table 2**).

#### Diagnostic tests

Amongst patients with a recorded diagnosis of heart failure in primary care, the use of diagnostic investigations increased substantially over time. In 2014, 82% of patients had at least one of the following tests: NP (8%), an ECG (37%), an echocardiogram (51%), or specialist assessment (28%), compared with 37% in 2002 (RR 2.24 [2.15, 2.34]) (**Table 2**).

#### Treatment initiation and dosages over time

Amongst patients with a diagnosis of heart failure recorded in primary care, reduced ejection fraction, and no contraindications or intolerances, prescriptions of essential drugs within three months of incident diagnosis differed by drug class. In 2014, prescription rates were high for ACE-Is or ARBs (80%) and beta blockers (72%) but lower for MRAs (28%) (**Table 2**).

Average daily doses prescribed over the 12 months following diagnosis remained below the recommended targets doses throughout the study period (2014 values: 42% for ACE-Is or ARBs, 29% for beta blockers, 22% for MRAs). When taken combined, the overall dose of beta blockers and ACE-Is/ARBs prescribed to patients changed little over time (adjusted difference from 2002 to 2014: +6% [+2%, +10%]), although patterns differed by individual drug classes and declined for ACE-Is or ARBs (adjusted difference from 2002 to 2014: −7% [−13%, −2%]) (**Table 2**).

**Fig 2**provides a graphical overview of care delivery indicators and diverging temporal trends.

### Treatment titration patterns before and after incident diagnosis

Prescription rates of the investigated drug classes in the three months preceding heart failure diagnosis were substantial (44% for ACE-Is or ARBs, 22% for beta blockers, and 3% for MRAs) and were largely related to an existing diagnosis of hypertension (RR for the prescription of at least one treatment class in patients with hypertension versus no hypertension: 2.07 [1.96, 2.18]).

**Fig 3:**
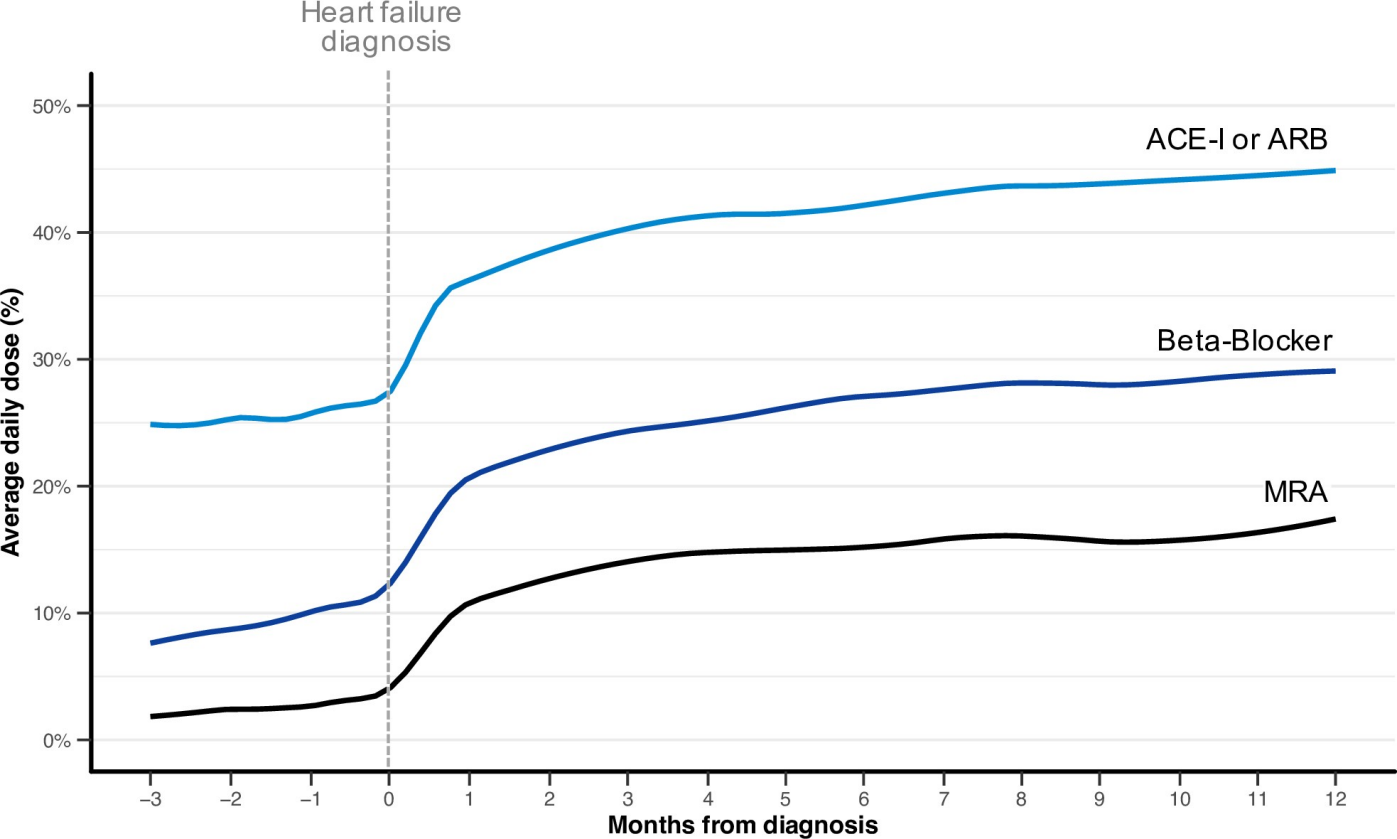
Average daily dose of guideline-recommended treatments prescribed around the time of incident heart failure in patients diagnosed from 2012–2014. Average daily dose prescribed to patients with heart failure and reduced ejection fraction without drug-specific contraindications or intolerances, from 3 months prior up to 12 months following incident heart failure, in patients diagnosed between 2012 and 2014, smoothed with local polynomial regression. Average daily dose is expressed as a percentage of the guideline-recommended target dose. ACE-I, angiotensin-converting–enzyme inhibitor; ARB, angiotensin receptor blocker; MRA, mineralocorticoid receptor antagonist.

**Fig 4:**
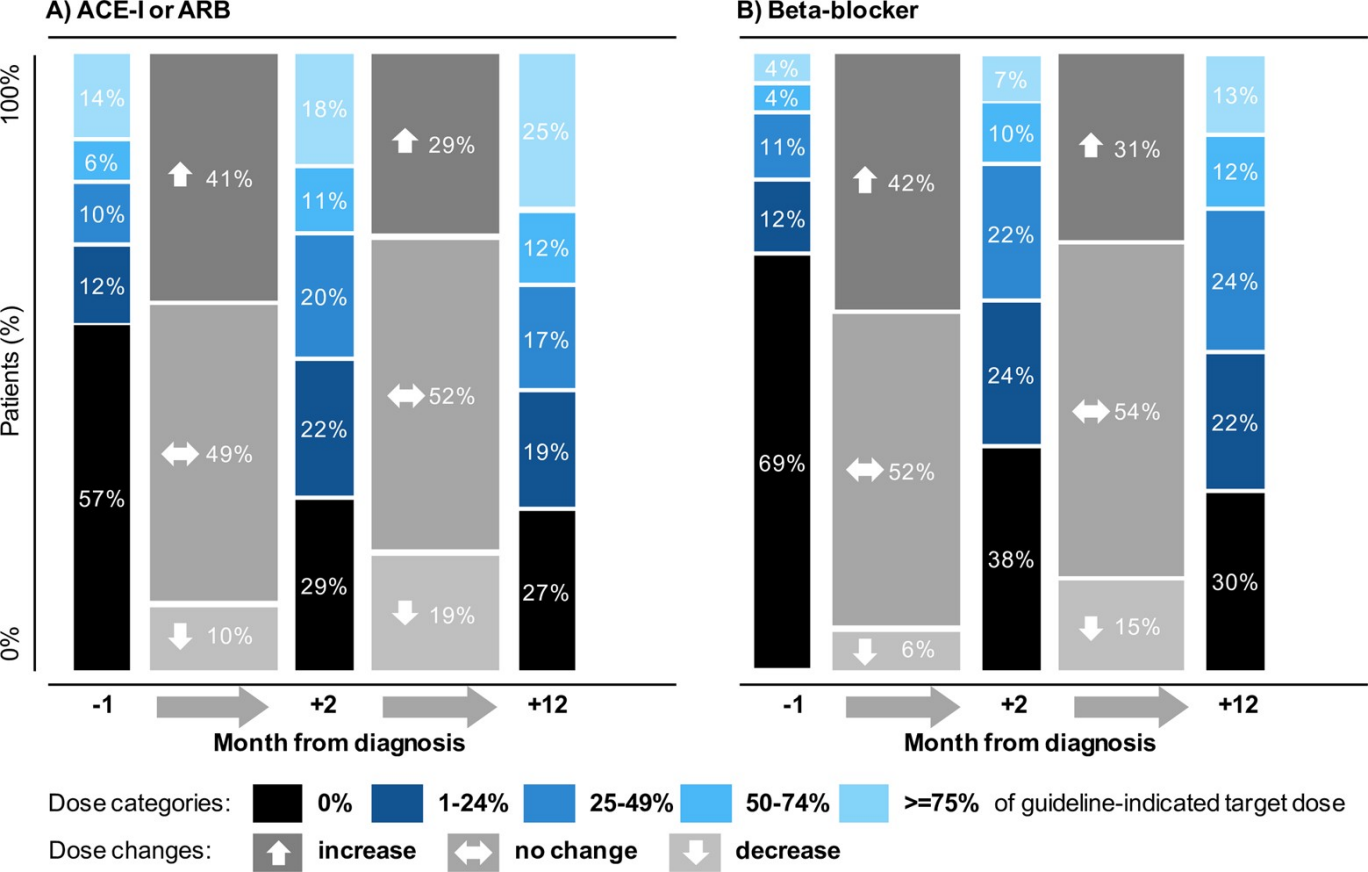
**Drug dose trajectory of (A) ACE-I or ARB dose and (B) beta blocker, prescribed around the time of incident heart failure, in patients diagnosed from 2012–2014.** Drug dose trajectory prescribed to patients with heart failure and reduced ejection fraction without contraindications or intolerances, in patients with incident heart failure between 2012 and 2014. ‘Month –1’ presents the average daily dose prescribed in the 30 days preceding incident heart failure. ‘Month 2’ presents the average daily dose prescribed in the second month (days 30 to 60) following incident heart failure. ‘Month 12’ presents the average daily dose prescribed in the twelfth month (days 335 to 365) following incident heart failure. ACE-I, angiotensin-converting–enzyme inhibitor; ARB, angiotensin receptor blocker.

Dose increments happened largely in the first 30 days following diagnosis, with no evidence of consistent increments thereafter (**Fig 3**). Because estimation of average doses across the whole population could potentially mask changes in dosing at the individual patient level, we further investigated changes to treatment dose by categories of ‘no change’, ‘increase’, or ‘decrease’ at fixed time intervals of 2 and 12 months after diagnosis. This showed that even in more recent years (2012–2014), for about half of the patients (52%), there was no change in dosage of ACE-Is or ARBs between month 2 and month 12 after diagnosis. The treatment dose decreased in 19% and increased in 29% of patients. At the end of one year, 27% of patients received no ACE-I or ARB treatment, and another 36% were on <50% of the guideline-recommended dose (**Fig 4A**). The patterns of changes were similar for beta blockers, for which a year after diagnosis, 30% did not receive this treatment (**Fig 4B**). Although overall doses changed over time, patterns of up-titration remained similar across time periods (**S1 Fig**).

To allow comparison with clinical trials and cross-sectional observational studies, we further report supplementary analyses on maximal prescribed doses as well as dosages amongst patients initiated on therapy (**S7 Table**).

#### Stratified analyses by age, sex, and socioeconomic status

**Fig 5:**
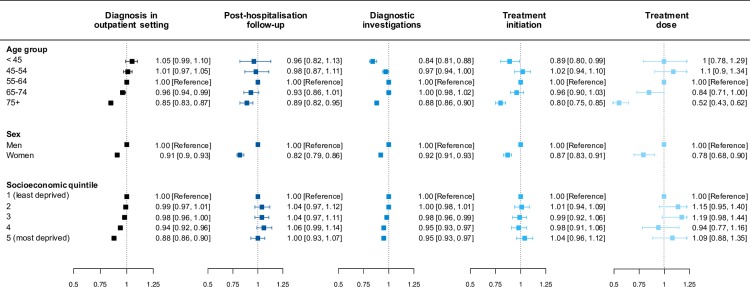
Stratified analysis of care delivery indicators by age, sex, and socioeconomic quintile in patients with heart failure in CPRD from 2002 to 2014. RRs and 95% CIs from multivariable Poisson regression model adjusted for year of diagnosis, age group, sex, socioeconomic status, and region. Diagnostic investigations refer to either one of echocardiogram, ECG, NPs, or specialist assessment, within ±6 months of incident heart failure diagnosis. Treatments refer to the prescription of both (i) ACE-Is or ARBs and (ii) BBs in patients with heart failure and reduced ejection fraction without recorded contraindications or intolerances. Treatment initiation indicates at least 1 prescription within 3 months of incident heart failure diagnosis. Treatment dose indicates whether a patients’ average daily dose, in the first 12 months following incident heart failure, was greater or equal to 50% of the guideline-recommended target dose. ACE-I, angiotensin-converting–enzyme inhibitor; ARB, angiotensin receptor blocker; BB, beta blocker; CI, confidence interval; CPRD, Clinical Practice Research Datalink; ECG, electrocardiogram; NP, natriuretic peptide; RR, risk ratio.

We observed variations in care by age, sex, and socioeconomic status (**Fig 5**).

Women were less commonly diagnosed in outpatient settings and had lower rates of primary care follow-up, fewer diagnostic investigations, less treatment initiation, and lower treatment doses than their male counterparts of similar age, socioeconomic status, and region.

Older patients (aged ≥75 years) exhibited similar and significant differences compared with their middle-aged counterparts (55 to 64 years old). The very young (<45 years) patients, particularly young women, had lower rates of diagnostic investigations across all four test types compared with the 55 to 64 years age group (**S8 Table**).

Amongst patients managed by their general practitioner, no socioeconomic disparities were apparent with regard to prescriptions of treatment or dose of treatment. However, deprived patients had lower rates of diagnosis in outpatient settings than their more affluent counterparts and hence were overall less likely to receive primary care follow-up.

Disparities remained apparent, although attenuated after adjusting for differences in follow-up times, 30-day mortality, and baseline comorbidities (**S2 Text**), with the exception of differences in primary care follow-up in patients aged ≥75 years.

## Discussion

This large-scale, population-based study provides important information on contemporary care of heart failure patients in routine clinical practice and insights into its variation over time by age, sex, and socioeconomic status. Our study confirms previous reports of high rates of guideline-indicated diagnostic investigations and treatment initiation in Western countries (**S1 Table**). However, further investigation of care across the continuum of primary and secondary services and from the prediagnosis stage to several months after incident diagnosis revealed important shortcomings in the management of patients. First, rates of outpatient diagnoses and follow-up in primary care after hospital discharge are low and have been declining over time. Second, doses of key medicines remain far below those recommended in guidelines in all groups of patients and for all three drug classes investigated, even a year after diagnosis. Finally, deficiencies in care were more common in women, older people, and, to some extent, socioeconomically deprived individuals.

Indeed, we found that rates of outpatient diagnoses have been declining whilst more patients are being diagnosed acutely in hospital. Relatedly, we found that only 17% of patients who were first diagnosed in hospital were subsequently followed up with a heart failure diagnosis in primary care, and this rate has also been declining, in particular when heart failure was not the primary discharge diagnosis. The reasons for the declining trends in diagnosis and follow-up of heart failure patients outside the acute hospital setting are not entirely clear and seem rather surprising when put in the context of the growing access to diagnostic services such as blood BNP outside hospitals and the reported shift in diagnosis from hospitals to outpatient settings elsewhere [21]. Our findings suggest that out-of-hospital screening and follow-up are suboptimal, and this could, at least partly, be due to poor record-keeping in primary care and inadequate information exchange between hospitals and primary care. It might also be that the national primary care reporting and incentive scheme [22] itself paradoxically contributed to these trends, with general practitioners, for instance, using free text descriptions to record patients’ problems rather than formally recording heart failure as a diagnosis. Such practices that avoid registering certain patients who will not achieve management recommendations to achieve higher overall adherence rates have been reported for other chronic conditions, such as depression [23,24]. Recent investments in specialist heart failure clinics and nurse-led services [25] might also have contributed to the observed patterns by creating artificially low primary care diagnosis and follow-up rates. Support for this comes from our sensitivity analyses, which indicate that some patients receive heart failure medicines in primary care despite no formal documentation of heart failure (**S1 Text**).

However, irrespective of the underlying reasons, the declining rates of heart failure recording in primary care are likely to have important consequences for patient care as well as research. The UK healthcare system relies on primary care as the cornerstone of chronic disease management. In such systems, general practitioners take a central role in screening, coordination, and continuous management of common conditions such as heart failure. Even where specialist clinics and nurse-led community services exist, general practitioners remain responsible for medication prescriptions and the coordination of specialty care. Accurate disease recording in primary care is therefore particularly important for this patient population with a high number of both cardiovascular and noncardiovascular comorbidities [15], and gaps in healthcare records are likely to have effects on subsequent monitoring and management of patients. This under-registration might also explain why some previous analyses concluded that the incidence of heart failure is declining in the UK [26] (which was refuted in subsequent studies that used linked records to capture cases across primary and secondary care) [15].

The increase in the proportion of patients initiated on evidence-based therapy for heart failure in recent years is important and encouraging because prescription of treatment, even at low dose, is an essential component of heart failure management. Yet gaps remain, particularly for beta blockers and MRAs. Whilst comparison across studies is difficult because of varying inclusion criteria and time points at which treatment plans are evaluated, rates appear somewhat lower than those reported in other countries (**S1 Table**), and underuse of these therapies are likely to be associated with significant loss of life, both quantity and quality [27]. Moreover, the average dose received per patient remained far below the guideline-recommended doses, and despite increasing numbers of patients initiated on therapy, the overall dose prescribed did not change substantially from 2002 to 2014. Titration was largely restricted to the 30-day period after incident diagnosis, yet landmark clinical trials have established the value of optimal drug dosages in conferring clinical benefits [28–30]. Whilst it may not be possible for every patient to achieve guideline-recommended doses, the rates reported by our study remain below rates achieved through community-based interventions or in clinical trials [31]. Our findings hence question whether current efforts in community heart failure services sufficiently address the long-term needs of patients with heart failure. In addition, they show that the current UK focus on monitoring diagnostic tests and treatment initiation might distract attention away from medication maintenance and titration as a measure of quality of care.

Our study further shows that the management of women, older people, and deprived individuals was even less satisfactory. For example, all were more likely to be first diagnosed during a hospital admission. This may suggest that, in these patients, early signs and symptoms have not been appropriately recognised in nonacute healthcare settings. Alternatively, this may be related to patient preferences to seek care in hospital or, for deprived populations, to more difficult access to outpatient consultations [32]. Correcting such disparities is an important challenge for a system that intends to offer equality of access to and quality of care.

Healthcare reporting efforts play an important role in improving quality of care. Our study presents changes in the delivery of care alongside the implementation of landmark quality improvement initiatives, in particular the QOF, a reporting and incentives scheme for primary care introduced in 2004 [22], and the NHFA, a reporting programme for secondary care introduced in 2007 [11]. These schemes represent the primary source of quality of care data in the UK so far and report a very positive picture of heart failure management. For example, both report very high rates of diagnostic investigations (91%–95%) [12,13] and treatment initiation (85%–99% for ACE-Is or ARBs) [12,13] that, given the small differences in variable definitions, are comparable to the present findings. However, the investigation of care across the continuum of primary and secondary services reveals important shortcomings in the management of patients that analyses confined to individual clinical settings are unable to identify. When reflecting specifically on the financial incentives programme, which applies to primary care, it appears that its design insufficiently addresses patients’ long-term care needs. The scheme incentivises general practitioners to follow evidence-based care practices and monitors over 100 indicators across a range of conditions. For heart failure, the scheme monitors referral for diagnostic investigations and yearly prescriptions of both beta blockers and ACE-Is or ARBs, with financial rewards only for primary care practices that achieve set target rates. Our findings reveal that incentivised indicators have improved over time but show no or negative changes in associated outcomes. Perhaps most importantly, they reveal possible unintended consequences in the fact that following the introduction of the programme, recording of diagnoses in primary care declined considerably, leading to a large and growing number of patients for whom continuous monitoring and management remains uncertain.

A major strength of this study is the use of a large population-based cohort of patients, which allows a sufficient number of cases in each age, sex, and socioeconomic category for subpopulation analyses and increases the generalisability of findings compared with surveys enrolling more selected participants. Moreover, longitudinal data from electronic health records provide a unique opportunity to follow patients over time in different care settings and hence to address the limitations of selected single-setting and single-time–point indicators used in previous studies. One of the key limitations of our study was the incomplete clinical information contained in the available electronic health records. In particular, left ventricular ejection fraction values were not available. Hence, we could only characterise the type of heart failure in a subset of patients for whom the diagnostic code made a clear reference to reduced ejection fraction. Whilst this subset of patients is likely to underestimate the true prevalence of reduced ejection fraction in the community, its high specificity ensures the selection of patients is appropriate for our analyses. Moreover, secondary care records did not provide access to diagnostic investigations, procedures (such as device implantations), discharge prescriptions or referrals, or outpatient consultations, so some analyses had to be restricted to primary care data. Finally, reliable information about patients’ symptoms was not available and limited our ability to investigate precise indications for certain therapies, such as MRAs.

Our findings have important implications for health services policies. The increased uptake of guideline-recommended diagnostic tests and treatment initiation amongst patients seen in primary care suggests that early management of these patients has improved, probably because of a combination of physician awareness, clinical guidelines, and financial incentives. However, the limited changes to medication dosages, the disparities amongst subgroups of patients, and the poor rates of primary care recording indicate that more efforts are needed. Quality improvement efforts that remained confined within individual care settings have proven insufficient to identify important care gaps and to address challenges of this chronic condition with effective but complex treatment. Further improvements are likely to require a broader perspective to health services design to support appropriate care at every level of the patient journey.

## Supporting information

S1 Text Documentation of diagnosis in primary care records as a prerequisite for subsequent disease monitoring and management. (DOCX)

S2 Text Method for the calculation of average daily doses. (DOCX)

S3 Text Sensitivity analyses. (DOCX)

S4 Text RECORD statement. RECORD, REporting of studies Conducted using Observational Routinely collected health Data.
(DOCX)

S5 Text Study design and analysis plan. (DOCX)

S1 Table Selected studies reporting care delivery in patients with heart failure. (DOCX)

S2 Table Clinical codes used to identify patients with heart failure and reduced ejection fraction from general practice records. (DOCX)

S3 Table Clinical codes used to identify drug-class–specific contraindications or intolerances from general practice records. (DOCX)

S4 Table Clinical codes used to identify diagnostic tests performed in patients with heart failure from general practice records. (DOCX)

S5 Table Recommended maintenance dosages of pharmacological treatments indicated in patients with heart failure and reduced ejection fraction, as per ESC and NICE guidelines during the study period. ESC, European Society of Cardiology; NICE, National Institute for Clinical Excellence.
(DOCX)

S6 Table Baseline characteristics of patients with incident heart failure by record of ejection fraction. (DOCX)

S7 Table Temporal trends in supplementary care indicators following incident heart failure by year of diagnosis. (DOCX)

S8 Table Diagnostic investigations following incident heart failure, stratified by age and sex. (DOCX)

S1 Fig Average daily dose of guideline-recommended treatments prescribed around the time of incident heart failure by time period of diagnosis. (DOCX)

## Abbreviations

ACE-I: angiotensin-converting-enzyme inhibitor
ARB: angiotensin receptor blocker
BMI: body-mass index
BNP: B-type natriuretic peptide
CI: confidence interval
CPRD: Clinical Practice Research Datalink
ECG: electrocardiogram
ESC: European Society of Cardiology
HES: Hospital Episodes Statistics
IMD: Index of Multiple Deprivation
ISAC: Independent Scientific Advisory Committee
MRA: mineralocorticoid receptor antagonist
NA: not applicable
NHFA: National Heart Failure Audit
NP: natriuretic peptide
NT-pro-BNP: N-terminal-pro-BNP
QOF: Quality and Outcomes Framework
RECORD: REporting of studies Conducted using Observational Routinely collected health Data
RR: risk ratio
