# Supplementary material for: Diagnostic tests, drug prescriptions, and follow-up patterns after incident heart failure: A cohort study of 93,000 UK patients
Source: PLoS Med. 2019 May 21;16(5):e1002805. doi: 10.1371/journal.pmed.1002805 (PMC6528949; doi:10.1371/journal.pmed.1002805)
Supplement: S1 Text — (DOCX) [file pmed.1002805.s001.docx]

S1 Text: Documentation of diagnosis in primary care records as a prerequisite for subsequent disease monitoring and management

In our main analyses, we assumed that the documentation of diagnosis in primary care records was a prerequisite for subsequent disease monitoring and management. Our rationale was that physicians are unlikely to initiate medications for conditions that are not formally diagnosed. To verify this assumption, we investigated pharmacological treatment among patients with heart failure recorded during a hospital admission, discharged alive, and with no documentation of heart failure in primary care. This revealed to be challenging, because hospital diagnoses are coded in the ICD-10 (International Classification of Diseases 10th edition) system which does not allow accurate identification of patients with reduced ejection fraction. Moreover, heart failure medicines are also used in the treatment of hypertension, which affects about two thirds of heart failure patients, and without a heart failure diagnosis one cannot reliably ascertain whether medications were given for heart failure or not. To best address the aforementioned circumstances, we investigated primary care rates of new prescriptions (initiation of therapy in patients who had not received these drugs in the 3 months prior to diagnosis) or dose increments (dose increase of 10% or more in patients who had received these drugs in the 3 months prior to diagnosis) for either ACE-I/ARB or beta-blocker within 3 months of diagnosis, and found these were present in 17% of patients. We considered extending the definition of follow-up in primary care with indications of pharmacological management and found that although this led to higher overall rates of follow-up in primary care (34% compared with 17% in our main analyses), temporal trends remained unchanged, i.e. declining by about 6% over the study period. We concluded that although there is evidence that some patients receive primary care follow-up despite no formal documentation of heart failure, these numbers were sufficiently low not to impact our main conclusions; and that our assumption was suitable for the purpose of this study.
