# Supplementary material for: Diagnostic tests, drug prescriptions, and follow-up patterns after incident heart failure: A cohort study of 93,000 UK patients
Source: PLoS Med. 2019 May 21;16(5):e1002805. doi: 10.1371/journal.pmed.1002805 (PMC6528949; doi:10.1371/journal.pmed.1002805)
Supplement: S2 Text — (DOCX) [file pmed.1002805.s002.docx]

S2 Text: Method for the calculation of average daily doses

For each prescription issued, we calculated *prescription duration* by dividing the prescribed quantity by the daily number of tablets. Prescriptions were considered active from the date they were recorded up to the end of their duration or a more recent prescription of the same drug class was made (whichever came first, so that at any time point a maximum of one prescription was valid). If on the same day two or more prescriptions were issued with the same duration and dosage, durations were aggregated. Further, if on the same day two or more prescriptions were issued with the same duration and different dosages, dosages were aggregated. If on the same day two or more prescriptions were issued with the different duration and dosages, the prescription with the strongest dose and then highest quantity was considered active.

For each prescription issued, we also calculated the *prescribed daily dose (PDD)* by multiplying the prescribed number of tablets per day and the tablet strengths in mg.

Finally, for every day that a patient was alive and registered with a participating general practice in the first 12 months following diagnosis, we calculated the *average daily dose (ADD)*.

The *average daily dose* was expressed as a fraction of the drug-specific guideline-recommended dose based on European Society of Cardiology guidelines for the treatment of heart failure effective during the study period (**table S3**), taking the lower target dose when a range was offered.
