# Supplementary material for: Diagnostic tests, drug prescriptions, and follow-up patterns after incident heart failure: A cohort study of 93,000 UK patients
Source: PLoS Med. 2019 May 21;16(5):e1002805. doi: 10.1371/journal.pmed.1002805 (PMC6528949; doi:10.1371/journal.pmed.1002805)
Supplement: S3 Text — (DOCX) [file pmed.1002805.s003.docx]

S3 Text: Sensitivity analyses

To confirm the robustness of our results, we performed the following sensitivity analyses:

**(a) accounting for patient-years of follow-up.** We computed rates as number of events / patient-years at risk, where time at risk was restricted to days alive, registered with a participating general practice, and to practice’s ‘up-to-standard' (UTS) periods. Rate ratios and 95% confidence intervals (CI) compare were computed using Poisson regression models with log offset of time-at-risk, adjusting for age, sex, socioeconomic status and region. These analyses led to higher rates of primary care follow-up, diagnostic investigations and treatment initiations, however temporal trends as well as disparities by age, sex, and socioeconomic status remained unchanged.

**(b) restricting analyses to those patients that were alive 30 days following their incident heart failure diagnosis**.

These analyses led to slightly higher rates of primary care follow-up, diagnostic investigations and treatment initiations, however temporal trends as well as disparities by age, sex, and socioeconomic status remained unchanged.

**(c) adjusting regression models for 17 comorbid conditions**. When adjusting regression models for 17 chronic conditions, we found that disparities by age, sex, and socioeconomic status were attenuated, nevertheless all reported disparities remained significant.

**(d) excluding those patients exempted from the national primary care audit ‘quality and outcomes framework’**.

Data from approximately 4% of patients with heart failure recorded in primary care, were exempted from the national primary care audit. Reasons for exemption included the labels ‘patient unsuitable’ and ‘informed dissent’. Excluding those patients from our analyses led to no significant changes in the present results.

**(e) restricting analyses to medicines recommended in the 2012 ESC guidelines**.

When restricting medicines to those recommended in the 2012 ESC guidelines, we found that rates of treatment initiation and dosages were slightly reduced (in the range of 3 to 5%). However, temporal trends as well as disparities by age, sex, and socioeconomic status remained unchanged.
