# Supplementary material for: Diagnostic tests, drug prescriptions, and follow-up patterns after incident heart failure: A cohort study of 93,000 UK patients
Source: PLoS Med. 2019 May 21;16(5):e1002805. doi: 10.1371/journal.pmed.1002805 (PMC6528949; doi:10.1371/journal.pmed.1002805)
Supplement: S5 Text — (DOCX) [file pmed.1002805.s005.docx]

# Quality of care in individuals with heart failure

Study design and analysis plan

##

## Background and rationale

Landmark clinical trials provide a strong evidence-base for the management of heart failure patients and demonstrate that appropriate treatment is effective in reducing death and hospitalisation rates.[1] However good clinical management of heart failure is complex and time consuming, and hence may be difficult to achieve in a general practice care delivery setting. Diagnostic and therapeutic guidelines (such as standards from the National Institute for Health and Clinical Excellence (NICE) first introduced in 2003[2] and updated in 2010[3] or guidelines from European Society of Cardiology (ESC) first introduced in 1995 and regularly updated subsequently[4–7]), as well as ‘pay-for-performance’ schemes (such as the Quality and Outcomes Framework (QOF) which started in 2004[8]), have therefore been progressively introduced seeking to improve evidence-based heart failure management in practice.

While several sources report a strong overall uptake of guideline-indicated therapies in many Western countries including the United Kingdom (UK),[9,10] a recent UK-based study reported no improvement in survival rates for patients diagnosed with heart failure in the community over the past two decades[11].

A comprehensive understanding of how care delivery components vary according to patient characteristics, such as age, sex, socio-economic status, region and associated co-morbidities, may help refine service delivery policies or prioritise further research, but current evidence base is limited.

## Objective and research questions

This study aims to analyse temporal trends and patterns in the quality of care received by heart failure patients in the United Kingdom, after incident heart failure, by important patient features such as age, sex, socio-economic status, region and co-morbidities.

To investigate the quality of care delivery, we chose to investigate three major components: diagnosis, treatment and care pathways. More specifically, we will investigate the proportion of (eligible) patients with incident heart failure who have undergone guideline-indicated diagnostic tests, received guideline-indicated treatments, and for whom diagnosis and follow-up was overseen by a general practitioner. We will further investigate temporal trends and patient-level factors explaining variation.

## Data Source

Linked primary and secondary health records from the Clinical Practice Research Datalink (CPRD), as per incidence analysis manuscript.

## Study population

Incident heart failure cases from 2002 to 2014, as per incidence analysis manuscript.[12]

## Case Identification

Case identification, as per incidence analysis manuscript. [12].

## Case categorization

We categorise cases as ‘HF with reduced ejection fraction’ (HF-REF) if codes make a clear reference to reduced ejection fraction within 1 year of their index diagnosis (n= 12,101, 13.0%).

## Baseline Variables

- Heart failure type (reduced or preserved ejection fraction)
- Age at heart failure diagnosis
- Socioeconomic status (patient-level IMD 2015 quintile)
- Sex
- Region
- Ethnicity
- Systolic and diastolic blood pressure (most recent measurement within 2 years prior to a diagnosis of heart failure)
- Body mass index (BMI) (most recent measurement within 2 years prior to a diagnosis of heart failure)
- Smoking status (most recent measurement within 2 years prior to a diagnosis of heart failure)
- Co-morbidities, prevalence of 17 common chronic conditions (anaemia, asthma, atrial fibrillation, cancer, chronic kidney disease, chronic obstructive pulmonary disease, dementia, depression, diabetes, dyslipidaemia, hypertension, ischaemic heart disease, obesity, osteoarthritis, peripheral arterial disease, stroke, thyroid disease) at any time prior to first diagnosis of heart failure in primary care or hospital discharge records
- Drug contra-indications and intolerances for each of (i) angiotensin-converting-enzyme inhibitors (ACE-I) or angiotensin receptor blockers (ARB); (ii) beta-blockers (BB); and (iii) mineralocorticoid receptor antagonists (MRA) (recorded at any point in time) defined as any drug-class-specific record of intolerance, allergy or patient refusal

## Outcome variables

**1.** **Diagnosis**: We will investigate referrals for the three diagnostic tests recommended as “essential” by the European Society of Cardiology (ESC) guidelines: (i) echocardiography; (ii) electrocardiogram; and (iii) natriuretic peptides, as well as specialist cardiology assessment, made within 3 months prior and 3 months after incident heart failure diagnosis.

This will lead to the following variables to be extracted from CPRD:

- echocardiography referral within 3 months prior and 12 months after incident heart failure diagnosis (dichotomous 1 / 0 variable)
- electrocardiogram referral within 3 months prior and 12 months after incident heart failure diagnosis (dichotomous 1 / 0 variable)
- natriuretic peptides referral within 3 months prior and 12 months after incident heart failure diagnosis (dichotomous 1 / 0 variable)
- specialist cardiology assessment referral within 3 months prior and 12 months after incident heart failure diagnosis (dichotomous 1 / 0 variable)
- diagnosis quality score**:** composite measure of diagnostic assessments, defined as appropriate if patient has either (a) a record of echocardiography, or (b) a record of a specialist cardiology assessment, (c) a record of a natriuretic peptide measurement and inappropriate in all other cases.

**2. Treatment:** We will investigate the prescriptions issued by general practitioners for the three drug classes indicated in the treatment of heart failure by the European Society of Cardiology (ESC) guidelines: (i) angiotensin-converting-enzyme inhibitors (ACE-I) or angiotensin receptor blockers (ARB); (ii) beta-blockers (BB); and (iii) mineralocorticoid receptor antagonists (MRA). For each of the three drug classes, we will investigate treatment initiation, measured as the issue of at least one prescription within 3 months of incident heart failure, as well as treatment maintenance measured as the average daily dose prescribed to eligible patients in the year following their first heart failure diagnosis, as a percentage of the guideline-indicated target dose.

Because drug treatment guideline recommendations exist only for patients with reduced ejection fraction, this analysis will be restricted to those patients categorised cases as ‘HF with reduced ejection fraction’ (HF-REF). Patients will be considered eligible if no contraindication or intolerance to that drug class was ever recorded.

**2.1 Treatment initiation**

Treatment initiation will be measured by the proportion of eligible patients who received at least 1 prescription within 3 months of their first heart failure diagnosis.

This will lead to the following variables to be extracted from CPRD:

- prescription of an angiotensin-converting-enzyme inhibitor (ACE-I) or angiotensin receptor blocker (ARB) within 1 month prior to 3 months after incident heart failure diagnosis (dichotomous 1 / 0 variable)
- prescription of a beta-blocker (BB) within 1 month prior to 3 months after incident heart failure diagnosis (dichotomous 1 / 0 variable)
- prescription of a mineralocorticoid receptor antagonist (MRA) within 1 month prior to 3 months after incident heart failure diagnosis (dichotomous 1 / 0 variable)
- treatment initiation quality score: sum of prescription initiation variables for ACE-I/ARB, BB and MRA, divided by 3.

**2.2 Treatment maintenance and up-titration**

Treatment maintenance and up-titration will be measured by the Average Daily Dose (ADD) prescribed to patients over all days a patient is alive in the first 12 months following their incident heart failure diagnosis. The average daily dose is calculated as the average prescribed daily dose, and expressed as a fraction of the drug-specific guideline-indicated dose (see **appendix 3** for details on drug dose extraction in CPRD). The guideline-indicated doses are defined as the minimal dosages recommended by the latest European Society of Cardiology guidelines for the treatment of heart failure available during the study period.

This will lead to the following variables to be extracted from CPRD:

- average daily dose of angiotensin-converting-enzyme inhibitors (ACE-I) or angiotensin receptor blockers (ARB) prescribed in the first 12 months after incident heart failure diagnosis, as a percentage of guideline-indicated target dose (continuous variable ranging from 0 to 1, computed for every month individually as well as aggregated over the 12 months)
- average daily dose of beta-blockers (BB) prescribed in the first 12 months after incident heart failure diagnosis, as a percentage of guideline-indicated target dose (continuous variable ranging from 0 to 1, computed for every month individually as well as aggregated over the 12 months)
- average daily dose of mineralocorticoid receptor antagonists (MRA) prescribed in the first 12 months after incident heart failure diagnosis, as a percentage of guideline-indicated target dose (continuous variable ranging from 0 to 1, computed for every month individually as well as aggregated over the 12 months)
- treatment maintenance quality score: sum of the average daily doses for ACE-I/ARB, BB and MRA, divided by 3.

**3.** **Care pathways**

We will investigate (i) the proportion of patients diagnosed in inpatient settings (primary or secondary hospital admission for heart failure) versus outpatient settings (primary care consultation), as well as (ii) the proportion of those patients diagnosed in hospital and discharged alive, who have received subsequent follow-up from their general practitioner in regard to their heart failure.

This will lead to the following variables to be extracted from CPRD/HES:

- record of diagnosis care setting (categorical variable with 3 levels)
- record of follow-up consultation, defined as a GP consultation with a record of heart failure within 6 months of the incident diagnosis (dichotomous 1 / 0 variable)

## Statistical Analysis

For each component, age-, sex-, SES-, region- and year-specific rates will be computed as the proportion of eligible patients (eg. patients for whom no intolerance/contra-indication is recorded) who received care within a defined time-frame of incident heart failure diagnosis.

Linear and Poisson regression models will be used to examine trends over time and by sub-groups. Corresponding 95% Confidence Intervals (CI) will be reported. Models will be adjusted for time, age, sex, region, socio-economic status, and blood pressure, where applicable.

We will further investigate the use of repeated measures models, such as generalized mixed-effects regression (MRM) models or generalized estimating equation (GEE) models, to examine temporal prescription patterns accounting for within-patient variability.

## Sensitivity analyses

To test the robustness of my results, I will restrict diagnostic codes to those used by the Quality of Outcomes Framework to assess the performance of heart failure care in the UK. I will examine whether this analysis leads to similar outcomes, and if not, investigate the reasons explaining the differences.

## Mock tables and figures

**Table 1**: Patient characteristics by sex, socio-economic status and time period

**Figure 1**: Graphical visualisation of overall care delivery in patients with heart failure

**Figure 2.1**: Temporal trends in diagnosis quality metrics by sex by calendar year from 2002 to 2014, with 5 panels: A) diagnosis quality score, B) echocardiography, C) specialist assessment, D) electrocardiogram, and (E) natriuretic peptides

**Figure 2.2**: Temporal trends in diagnosis quality metrics by socio-economic-status by calendar year from 2002 to 2014, with 5 panels: A) diagnosis quality score, B) echocardiography, C) specialist assessment, D) electrocardiogram, and (E) natriuretic peptides

**Figure 2.3**: Temporal trends in diagnosis quality metrics by region by calendar year from 2002 to 2014, with 5 panels: A) diagnosis quality score, B) echocardiography, C) specialist assessment, D) electrocardiogram, and (E) natriuretic peptides

**Figure 2.4**: Temporal trends in diagnosis quality metrics by diagnosis setting (acute vs non-acute) by calendar year from 2002 to 2014, with 5 panels: A) diagnosis quality score, B) echocardiography, C) specialist assessment, D) electrocardiogram, and (E) natriuretic peptides

**Figure 3.1**: Temporal trends in treatment initiation quality metrics by sex by calendar year from 2002 to 2014, with 4 panels: A) treatment initiation quality score, B) angiotensin-converting-enzyme inhibitors (ACE-I) or angiotensin receptor blockers (ARB) initiation, C) beta-blockers (BB) initiation, D) mineralocorticoid receptor antagonists (MRA) initiation

**Figure 3.2**: Temporal trends in treatment initiation quality metrics by socio-economic-status by calendar year from 2002 to 2014, with 4 panels: A) treatment initiation quality score, B) angiotensin-converting-enzyme inhibitors (ACE-I) or angiotensin receptor blockers (ARB) initiation, C) beta-blockers (BB) initiation, D) mineralocorticoid receptor antagonists (MRA) initiation

**Figure 3.3**: Temporal trends in treatment initiation quality metrics by region by calendar year from 2002 to 2014, with 4 panels: A) treatment initiation quality score, B) angiotensin-converting-enzyme inhibitors (ACE-I) or angiotensin receptor blockers (ARB) initiation, C) beta-blockers (BB) initiation, D) mineralocorticoid receptor antagonists (MRA) initiation

**Figure 3.4**: Temporal trends in treatment initiation quality metrics by diagnosis setting (acute vs non-acute) by calendar year from 2002 to 2014, with 4 panels: A) treatment initiation quality score, B) angiotensin-converting-enzyme inhibitors (ACE-I) or angiotensin receptor blockers (ARB) initiation, C) beta-blockers (BB) initiation, D) mineralocorticoid receptor antagonists (MRA) initiation

**Figure 4.1**: Temporal trends in treatment maintenance quality metrics by sex by calendar year from 2002 to 2014, with 4 panels: A) treatment maintenance quality score, B) angiotensin-converting-enzyme inhibitors (ACE-I) or angiotensin receptor blockers (ARB) maintenance, C) beta-blockers (BB) maintenance, D) mineralocorticoid receptor antagonists (MRA) maintenance

**Figure 4.2**: Temporal trends in treatment maintenance quality metrics by socio-economic-status by calendar year from 2002 to 2014, with 4 panels: A) treatment maintenance quality score, B) angiotensin-converting-enzyme inhibitors (ACE-I) or angiotensin receptor blockers (ARB) maintenance, C) beta-blockers (BB) maintenance, D) mineralocorticoid receptor antagonists (MRA) maintenance

**Figure 4.3**: Temporal trends in treatment maintenance quality metrics by region by calendar year from 2002 to 2014, with 4 panels: A) treatment maintenance quality score, B) angiotensin-converting-enzyme inhibitors (ACE-I) or angiotensin receptor blockers (ARB) maintenance, C) beta-blockers (BB) maintenance, D) mineralocorticoid receptor antagonists (MRA) maintenance

**Figure 4.4**: Temporal trends in treatment maintenance quality metrics by diagnosis setting (acute vs non-acute) by calendar year from 2002 to 2014, with 4 panels: A) treatment maintenance quality score, B) angiotensin-converting-enzyme inhibitors (ACE-I) or angiotensin receptor blockers (ARB) maintenance, C) beta-blockers (BB) maintenance, D) mineralocorticoid receptor antagonists (MRA) maintenance

**Figure 5**: Treatment maintenance pattern in patients with incident heart failure, in the first 12 months following incident heart failure, with 4 panels: A) treatment maintenance quality score, B) angiotensin-converting-enzyme inhibitors (ACE-I) or angiotensin receptor blockers (ARB) maintenance, C) beta-blockers (BB) maintenance, D) mineralocorticoid receptor antagonists (MRA) maintenance

## References

1. Sacks CA, Jarcho JA, Curfman GD. Paradigm Shifts in Heart-Failure Therapy — A Timeline Paradigm Shifts in Heart-Failure Therapy. n engl j med. 2014;37111. Available: http://www.nejm.org/doi/pdf/10.1056/NEJMp1410241

2. National Institute for Clinical Excellence (NICE). Management of chronic heart failure in adults in primary and secondary care. Clinical Guideline 5. 2003.

3. National Institute for Health and Clinical Excellence (NICE). Chronic heart failure: management of chronic heart failure in adults in primary and secondary care (CG108) [Internet]. 2010. Available: http://guidance.nice.org.uk/CG108/Guidance

4. Remme WJ, Swedberg K. Task Force Report Guidelines for the diagnosis and treatment of chronic heart failure Diagnosis of chronic heart failure. Eur Heart J. 2001;22: 1527–1560. doi:10.1053/euhj.2001.2783

5. Swedberg K, Cleland J, Dargie H, Drexler H, Follath F, Komajda M, et al. Guidelines for the diagnosis and treatment of chronic heart failure: executive summary (update 2005): The Task Force for the Diagnosis and Treatment of Chronic Heart Failure of the European Society of Cardiology. Eur Heart J. Oxford University Press; 2005;26: 1115–1140. doi:10.1093/eurheartj/ehi204

6. Dickstein K, Cohen-Solal A, Filippatos G, McMurray JJ, Ponikowski P, Poole-Wilson PA, et al. ESC guidelines for the diagnosis and treatment of acute and chronic heart failure 2008: the Task Force for the diagnosis and treatment of acute and chronic heart failure 2008 of the European Society of Cardiology. Developed in collaboration with the Heart. Eur J Hear Fail. 2008/10/02. 2008;10: 933–989. doi:S1388-9842(08)00370-X [pii] 10.1016/j.ejheart.2008.08.005

7. McMurray JJ V, Adamopoulos S, Anker SD, Auricchio A, Böhm M, Dickstein K, et al. ESC Guidelines for the diagnosis and treatment of acute and chronic heart failure 2012: The Task Force for the Diagnosis and Treatment of Acute and Chronic Heart Failure 2012 of the European Society of Cardiology. Developed in collaboration with the Heart. Eur Heart J. 2012;33: 1787–847. doi:10.1093/eurheartj/ehs104

8. Health and Social Care Information Centre. National Quality and Outcomes Framework Statistics for England 2004/05 [Internet]. 2005. Available: http://content.digital.nhs.uk/catalogue/PUB01946/qof-eng-04-05-rep.pdf

9. Mitchell P, Marle D, Donkor A, Shote A, McDonagh T, Hardman S, et al. National Heart Failure Audit 2013-2014 [Internet]. 2015. Available: https://www.ucl.ac.uk/nicor/audits/heartfailure/documents/annualreports/hfannual13-14-updated.pdf

10. Primary Care Domain ND. Quality and Outcomes Framework Report, England 2015-16 [Internet]. 2016. Available: http://content.digital.nhs.uk/qof

11. Taylor CJ, Ryan R, Nichols L, Gale N, Hobbs R, Marshall T. Survival following a diagnosis of heart failure in primary care. Fam Pract. 2017; 1–8. doi:10.1093/fampra/cmw145

12. Conrad N, Judge A, Tran J, Mohseni H, Hedgecott D, Crespillo AP, et al. Temporal trends and patterns in heart failure incidence: a population-based study of 4 million individuals. Lancet. Elsevier; 2017;391: 572–580. doi:10.1016/S0140-6736(17)32520-5
