# Supplementary material for: Diagnostic tests, drug prescriptions, and follow-up patterns after incident heart failure: A cohort study of 93,000 UK patients
Source: PLoS Med. 2019 May 21;16(5):e1002805. doi: 10.1371/journal.pmed.1002805 (PMC6528949; doi:10.1371/journal.pmed.1002805)
Supplement: S1 Table — (DOCX) [file pmed.1002805.s006.docx]

S1 Table: Selected studies reporting care delivery in patient with heart failure.

| **Study name** | **Publication year** | **Country** | **Data collection period** | **Size** | **Design** | **Case identification** | **Assessment time points** | **Prevalent vs. incident cases** | **Type of heart failure** | **Main measures and results** |
| --- | --- | --- | --- | --- | --- | --- | --- | --- | --- | --- |
| BIOSTAT-CHF[1] | 2017 | 11 European countries | 2010-2012 | 2,100 | Prospective registry | Inpatients or outpatient clinics | Three months following registry enrolment | Prevalent | HF-REF or HF with abnormal BNP levels | **Up-titration to target dose** (at 3 months)  ACE-I or ARB (22%)  Beta-blocker (12%) |
| QUALIFY[2] | 2016 | 36 countries | 2013-2014 | 7,092 | Prospective registry | Inpatients or outpatient clinics | On registry enrolment | Prevalent | HF-REF | **Treatment prescription** (on enrolment)  ACE-I (66%) \| ARB (22%)  Beta-blocker (87%)  MRA (69%)  Ivabradine (33%)  **Up-titration to target dose** (on enrolment)  ACE-I (28%) \| ARB (7%)  Beta-blocker (15%)  MRA (71%)  Ivabradine (27%) |
| ESC-HF-LT[3] | 2016 | 21 European and/or Mediterranean countries | 2011 - 2013 | 12 440 | Prospective registry | Inpatients or outpatient clinics | On registry enrolment and at 1-year post enrolment | Prevalent | Any heart failure | **Treatment prescription** (on enrolment \| at 1 year)  ACE-I or ARB (89% \| 87%)  Beta-blocker (89% \| 89%)  MRA (59% \| 59%)  Diuretics (83% \| 81%) |
| Austrian Heart Failure Registry[4] | 2014 | Austria | 2006–2010 | 1,014 | Prospective registry | Outpatient heart failure clinics | On registry enrolment and at 1-year post enrolment | Prevalent | HF-REF | **Treatment prescription** (on enrolment \| at 1 year)  ACE-I or ARB (91% \| 92%)  Beta-blocker at baseline (88% \| 92%)  MRA at baseline (43% \| 39%)  **Treatment up-titration**  Patients achieving a dose of ≥50% of target dose for ACE-I/ARB and BB at one-year follow-up (64%) |
| QOF[5] | 2014 | England | 2013-2014 | 405,202 (HF) | Retrospective population-based cohort | Primary care centres | Within -3 and +12 months of incident diagnosis | Incident | Any heart failure | **Diagnostic investigations** (within -3 and +12 months of diagnosis)  Echocardiography or specialist assessment (95%) |
|  |  |  |  | 119,944 (HF-REF) |  |  | By financial year | Prevalent |  | **Treatment prescription** (yearly assessment)  ACE-I or ARB (99%)  Beta-blocker (93%) |
| Hawkins et al.[6] | 2012 | UK | 1999-2007 | 13,330 | Retrospective population-based cohort | Primary care centres | By calendar year | Prevalent | Any heart failure | **Treatment prescription** (in 2007)  ACE-I or ARB (64%)  Beta-blocker (41%)  Spironolactone (20%) |
| HELUMA[7] | 2010 | Germany | 2001-2007 | 3,292 | Prospective registry | Outpatient cardiology clinics or hospital admissions | On registry enrolment | Prevalent | Chronic heart failure due to systolic dysfunction | **Treatment prescription** (on enrolment)  ACE-I or ARB (87%)  Beta-blocker (87%)  MRA (49%)  **Treatment dose** (% of target on enrolment)  ACE-I or ARB (55%)  Beta-blocker (56%) |
| Swedish Heart Failure Registry[8] | 2009 | Sweden | 2004-2006 | 2,093 | Retrospective registry | Primary care centres | On registry enrolment | Prevalent | Any heart failure, with pharmacological treatment for heart failure | **Diagnostic investigations** (on enrolment)  Echocardiography (31%)  **Treatment prescription** (on enrolment)  ACE-I or ARB (74%)  Beta-blocker (67%)  **Up-titration >=50% of target dose** (on enrolment)  ACE-I or ARB (37%)  Beta-blocker (31%) |
| IMPROVE HF^15^ | 2008 | USA | 2005-2007 | 15,381 | Prospective registry | Outpatient clinics | On registry enrolment | Prevalent | HF-REF | **Treatment prescription** (on enrolment)  ACE-I or ARB (80%)  Beta-blocker (86%)  MRA (36%)  Anticoagulant (69%)  ICD/CRT-D (51%)  CRT (39%)  Patient education (61%) |
| MAHLER[11] | 2005 | 6 European countries | 2001-2002 | 1,410 | Prospective registry | Outpatient clinics | On registry enrolment and at 6-month post-enrolment | Prevalent | Any heart failure | **Treatment prescription** (at 6 months)  ACE-I or ARB (85%)  Diuretics (83%)  Beta-blocker (58%)  Spironolactone (36%)  Glycosides (52%) |

**Abbreviations**: HF = Heart failure; HF-REF = Heart failure and reduced ejection fraction; ACE-I = angiotensin-converting-enzyme inhibitor; ARB = angiotensin receptor blocker; MRA = Mineralocorticoid receptor antagonists; ICD = implantable cardioverter defibrillator; CRT-D = cardiac resynchronization therapy with defibrillator; CRT = cardiac resynchronization therapy.

**References**:

1. Ouwerkerk W, Voors AA, Anker SD, Cleland JG, Dickstein K, Filippatos G, et al. Determinants and clinical outcome of uptitration of ACE-inhibitors and beta-blockers in patients with heart failure: a prospective European study. Eur Heart J. Springer International Publishing, Cham; 2017;38: 1883–1890. doi:10.1093/eurheartj/ehx026

2. Komajda M, Anker SD, Cowie MR, Filippatos GS, Mengelle B, Ponikowski P, et al. Physicians’ adherence to guideline-recommended medications in heart failure with reduced ejection fraction: data from the QUALIFY global survey. Eur J Heart Fail. John Wiley & Sons, Ltd; 2016;18: 514–522. doi:10.1002/ejhf.510

3. Crespo-Leiro MG, Anker SD, Maggioni AP, Coats AJ, Filippatos G, Ruschitzka F, et al. European Society of Cardiology Heart Failure Long-Term Registry (ESC-HF-LT): 1-year follow-up outcomes and differences across regions. Eur J Heart Fail. John Wiley & Sons, Ltd; 2016;18: 613–625. doi:10.1002/ejhf.566

4. Poelzl G, Altenberger J, Pacher R, Ebner C h., Wieser M, Winter A, et al. Dose matters! Optimisation of guideline adherence is associated with lower mortality in stable patients with chronic heart failure. Int J Cardiol. 2014;175: 83–89. doi:10.1016/j.ijcard.2014.04.255

5. Health and Social Care Information Centre. Prevalence, Achievements and Exceptions Report from the Quality and Outcomes Framework, England 2013-14 [Internet]. 2014. Available: http://content.digital.nhs.uk/catalogue/PUB15751/qof-1314-report-V1.1.pdf

6. Hawkins NM, Scholes S, Bajekal M, Love H, O’Flaherty M, Raine R, et al. Community care in England: reducing socioeconomic inequalities in heart failure. Circulation. 2012/07/28. 2012;126: 1050–1057. doi:10.1161/CIRCULATIONAHA.111.088047

7. Frankenstein L, Remppis A, Fluegel A, Doesch A, Katus HA, Senges J, et al. The association between long-term longitudinal trends in guideline adherence and mortality in relation to age and sex. Eur J Heart Fail. 2010;12: 574–580. doi:10.1093/eurjhf/hfq047

8. Dahlstrom U, Hakansson J, Swedberg K, Waldenstrom A. Adequacy of diagnosis and treatment of chronic heart failure in primary health care in Sweden. Eur J Heart Fail. 2009;11: 92–98. doi:10.1093/eurjhf/hfn006

9. Fonarow GC, Yancy CW, Albert NM, Curtis AB, Stough WG, Gheorghiade M, et al. Heart failure care in the outpatient cardiology practice setting: findings from IMPROVE HF. Circ Heart Fail. American Heart Association, Inc.; 2008;1: 98–106. doi:10.1161/CIRCHEARTFAILURE.108.772228

10. Fonarow GC, Albert NM, Curtis AB, Stough WG, Gheorghiade M, Heywood JT, et al. Improving evidence-based care for heart failure in outpatient cardiology practices: primary results of the Registry to Improve the Use of Evidence-Based Heart Failure Therapies in the Outpatient Setting (IMPROVE HF). Circulation. 2010/07/28. 2010;122: 585–596. doi:CIRCULATIONAHA.109.934471 [pii]10.1161/CIRCULATIONAHA.109.934471

11. Komajda M, Lapuerta P, Hermans N, Gonzalez-Juanatey JR, van Veldhuisen DJ, Erdmann E, et al. Adherence to guidelines is a predictor of outcome in chronic heart failure: the MAHLER survey. Eur Heart J. Oxford University Press; 2005;26: 1653–1659. doi:10.1093/eurheartj/ehi251
