# Supplementary material for: Diagnostic tests, drug prescriptions, and follow-up patterns after incident heart failure: A cohort study of 93,000 UK patients
Source: PLoS Med. 2019 May 21;16(5):e1002805. doi: 10.1371/journal.pmed.1002805 (PMC6528949; doi:10.1371/journal.pmed.1002805)
Supplement: S2 Table — (DOCX) [file pmed.1002805.s007.docx]

S2 Table: Clinical codes used to identify patients with heart failure and reduced ejection fraction

from general practice records

| **Medcode** | | | **Read Code** | | **Description** | |  |
| --- | --- | --- | --- | --- | --- | --- | --- |
| 8966 | | | G5yy900 | | Left Ventricular Systolic Dysfunction | |  |
| 11284 | | | 585f.00 | | Echocardiogram shows left ventricular systolic dysfunction | |  |
| 5942 | | | G581.13 | | Impaired left ventricular function | |  |
| 7251 | | | 33BA.00 | | Impaired Left Ventricular Function | |  |
| 8010 | | | G551.00 | | Hypertrophic obstructive cardiomyopathy | |  |
| 11351 | | | 585g.00 | | Echo shows LVDD | |  |
| 7535 | | | G554400 | | Primary dilated cardiomyopathy | |  |
| 3499 | | | G554300 | | Hypertrophic non-obstructive cardiomyopathy | |  |
| 7320 | | | G343.00 | | Ischaemic cardiomyopathy | |  |
| 107397 | | | G5yyD00 | | Left ventricular cardiac dysfunction | |  |
| 4915 | | | G555.00 | | Alcoholic cardiomyopathy | |  |
| 9402 | | | G55y.11 | | Secondary dilated cardiomyopathy | |  |
| 97780 | | | G559.00 | | Arrhythmogenic right ventricular cardiomyopathy | |  |
| 70648 | | | Gyu5M00 | | [X]Other hypertrophic cardiomyopathy | |  |
|  |  | |  | |  |  |  |
