# Supplementary material for: Diagnostic tests, drug prescriptions, and follow-up patterns after incident heart failure: A cohort study of 93,000 UK patients
Source: PLoS Med. 2019 May 21;16(5):e1002805. doi: 10.1371/journal.pmed.1002805 (PMC6528949; doi:10.1371/journal.pmed.1002805)
Supplement: S3 Table — (DOCX) [file pmed.1002805.s008.docx]

S3 Table: Clinical codes used to identify drug-class-specific contraindications or intolerances

from general practice records

| Drug Class | **Medcode** | **Read code** | **Description** |
| --- | --- | --- | --- |
| ACE inhibitors | 13043 | 14LM.00 | H/O: angiotensin converting enzyme inhibitor allergy |
| ACE inhibitors | 11130 | U60C400 | [X]Angiotensin-convert-enz inhib caus advers eff therap use |
| ACE inhibitors | 26495 | U60C414 | [X]Adverse reaction to lisinopril |
| ACE inhibitors | 45023 | U60C412 | [X] Adverse reaction to enalapril |
| ACE inhibitors | 47141 | U60C413 | [X] Adverse reaction to ramipril |
| ACE inhibitors | 68465 | U60C411 | [X] Adverse reaction to captopril |
| ACE inhibitors | 48350 | TJC7700 | Adverse reaction to captopril |
| ACE inhibitors | 20747 | TJC7900 | Adverse reaction to ramipril |
| ACE inhibitors | 21039 | TJC7800 | Adverse reaction to enalapril |
| ACE inhibitors | 25369 | ZV14D00 | [V]PH angiotensin-converting-enzyme inhibitor allergy |
| ACE inhibitors | 104857 | K043000 | Acute renal failure due to ACE inhibitor |
| ACE inhibitors | 25369 | ZV14D00 | [V]PH angiotensin-converting-enzyme inhibitor allergy |
| ACE inhibitors | 10568 | 8I28.00 | Angiotensin converting enzyme inhibitors contraindicated |
| ACE inhibitors | 12135 | 8I3D.00 | Angiotensin converting enzyme inhibitor declined |
| ACE inhibitors | 10883 | 8I64.00 | Angiotensin converting enzyme inhibitor not indicated |
| ACE inhibitors | 11350 | 8I74.00 | Angiotensin converting enzyme inhibitor not tolerated |
| ARB | 13040 | 14LN.00 | H/O: angiotensin II receptor antagonist allergy |
| ARB | 11601 | U60CB00 | [X]Angiotensin II receptor antagon adverse effect therap use |
| ARB | 34469 | ZV14E00 | [V]PH of angiotensin II receptor antagonist allergy |
| ARB | 11510 | 8I2H.00 | Angiotensin II receptor antagonists contraindicated |
| ARB | 22335 | 8I3P.00 | Angiotensin II receptor antagonist declined |
| ARB | 18721 | 8I6C.00 | Angiotensin II receptor antagonist not indicated |
| ARB | 12571 | 8I75.00 | Angiotensin II receptor antagonist not tolerated |
| Beta Blocker | 13033 | 14LL.00 | H/O: betablocker allergy |
| Beta Blocker | 11667 | TJC6.00 | Adverse reaction to betablockers |
| Beta Blocker | 98477 | U60B900 | [X]Adverse reaction to bisoprolol |
| Beta Blocker | 98766 | U60BB00 | [X]Adverse reaction to nebivolol |
| Beta Blocker | 100019 | ZVu6i00 | [X]Personal history of allergy to bisoprolol |
| Beta Blocker | 47554 | ZV14C00 | [V]Personal history of betablocker allergy |
| Beta Blocker | 68505 | TJC0000 | Adverse reaction to practolol |
| Beta Blocker | 46411 | TJC0200 | Adverse reaction to propranolol |
| Beta Blocker | 10567 | 8I26.00 | Beta blocker contraindicated |
| Beta Blocker | 98220 | 8I2g.00 | Bisoprolol contraindicated |
| Beta Blocker | 98752 | 8I2h.00 | Carvedilol contraindicated |
| Beta Blocker | 100018 | 8I2i.00 | Nebivolol contraindicated |
| Beta Blocker | 10767 | 8I36.00 | Beta blocker therapy refused |
| Beta Blocker | 98478 | 8IAS.00 | Bisoprolol therapy refused |
| Beta Blocker | 100020 | 8IAT.00 | Carvedilol therapy refused |
| Beta Blocker | 100021 | 8IAV.00 | Nebivolol therapy refused |
| Beta Blocker | 10566 | 8I62.00 | Beta blocker not indicated |
| Beta Blocker | 98360 | 8I6i.00 | Bisoprolol not indicated |
| Beta Blocker | 99741 | 8I6j.00 | Carvedilol not indicated |
| Beta Blocker | 99742 | 8I6k.00 | Nebivolol not indicated |
| Beta Blocker | 11231 | 8I73.00 | Beta blocker not tolerated |
| Beta Blocker | 98301 | 8I7K.00 | Bisoprolol not tolerated |
| Beta Blocker | 100199 | 8I7L.00 | Carvedilol not tolerated |
| Beta Blocker | 100200 | 8I7M.00 | Nebivolol not tolerated |
| Beta Blocker | 13026 | TJC6200 | Adverse reaction to atenolol |
| Beta Blocker | 39564 | TJC6700 | Adverse reaction to sotalol |
| Beta Blocker | 42993 | TJC6z00 | Adverse reaction to betablockers NOS |
| Beta Blocker | 43499 | TJC6100 | Adverse reaction to acebutolol |
| Beta Blocker | 46076 | TJC6400 | Adverse reaction to metoprolol |
| Beta Blocker | 52662 | TJC6800 | Adverse reaction to timolol |
| Beta Blocker | 57521 | TJC6300 | Adverse reaction to labetalol |
| Beta Blocker | 61975 | TJC6500 | Adverse reaction to nadolol |
| Beta Blocker | 94109 | TJC6600 | Adverse reaction to oxprenolol |
| Beta Blocker | 20916 | U60B711 | [X] Adverse reaction to betablockers |
| Beta Blocker | 30300 | U60B700 | [X]Beta-adrenorecep antag caus advers eff in ther use, NEC |
| Beta Blocker | 41412 | U60B712 | [X] Adverse reaction to propranolol |
| Beta Blocker | 46541 | U60B71C | [X] Adverse reaction to betablockers NOS |
| Beta Blocker | 48351 | U60B715 | [X] Adverse reaction to atenolol |
| Beta Blocker | 49679 | U60B71A | [X] Adverse reaction to sotalol |
| Beta Blocker | 65814 | U60B716 | [X] Adverse reaction to labetolol |
| Beta Blocker | 73878 | U60B717 | [X] Adverse reaction to metoprolol |
| Beta Blocker | 96531 | U60B714 | [X] Adverse reaction to acebutolol |
| Beta Blocker | 109335 | U60B71B | [X] Adverse reaction to timolol |
| Beta Blocker | 98765 | U60BA00 | [X]Adverse reaction to carvedilol |
| MR Antagonist | 48902 | 8I2L.00 | Spironolactone contraindicated |
| MR Antagonist | 50174 | TJE4400 | Adverse reaction to spironolactone |
| MR Antagonist | 55032 | U60E51C | [X] Adverse reaction to spironolactone |
| MR Antagonist | 100950 | 8I3K000 | Spironolactone declined |
| MR Antagonist | 110297 | 8I6A000 | Spironolactone not indicated |
| MR Antagonist | 110011 | 8I2D000 | Eplerenone contraindicated |
| MR Antagonist | 110032 | 8I3K100 | Eplerenone therapy declined |

***Abbreviations****: ACE = angiotensin-converting enzyme; ARB = angiotensin receptor blocker; MRA = mineralocorticoid receptor antagonist; NICE =* National Institute for Health and Clinical Excellence.
