# Supplementary material for: Diagnostic tests, drug prescriptions, and follow-up patterns after incident heart failure: A cohort study of 93,000 UK patients
Source: PLoS Med. 2019 May 21;16(5):e1002805. doi: 10.1371/journal.pmed.1002805 (PMC6528949; doi:10.1371/journal.pmed.1002805)
Supplement: S4 Table — (DOCX) [file pmed.1002805.s009.docx]

S4 Table: Clinical codes used to identify diagnostic tests performed in patients with heart failure

from general practice records

To compile the list of diagnostic codes, we first extracted all codes in the code dictionary that referred to the description and synonyms of the procedure of interest; we then complemented that list with those codes used in the UK’s general practice pay for performance scheme (the ‘quality and outcomes framework’); finally, a cardiologist manually reviewed all codes and selected those that appropriately referred to the diagnostic procedure of interest.

**A. Echocardiogram**

| **Code Type** | | **Code** | |  | | **Description** |
| --- | --- | --- | --- | --- | --- | --- |
| Entity | | 342 | | Echocardiogram | |  |
| Read | | 5853.11 | | Echocardiogram | |  |
| Read | | 7935200 | | Transoesophageal echocardiography | |  |
| Read | | 5853100 | | Echocardiogram abnormal | |  |
| Read | | R132000 | | [D]Echocardiogram abnormal | |  |
| Read | | 585f.00 | | Echocardiogram shows left ventricular systolic dysfunction | |  |
| Read | | 585g.00 | | Echocardiogram shows left ventricular diastolic dysfunction | |  |
| Read | | 5853 | | U-S heart scan | |  |
| Read | | 5853z00 | | U-S heart scan NOS | |  |
| Read | | 5C20.00 | | Echocardiogram equivocal | |  |
| Read | | 33BB.00 | | Left ventricular ejection fraction | |  |
| Read | | 7P0H100 | | Transoesophageal echocardiography | |  |
| Read | | 7P0H000 | | Transthoracic echocardiography | |  |
| Read | | 7P0H400 | | Stress echocardiography | |  |
| Read | | 7935500 | | Transluminal intracardiac echocardiography | |  |
| Read | | 7P0Hz00 | | Diagnostic echocardiography NOS | |  |
| Read | | 7P0H300 | | Epicardial echocardiography | |  |
| Read | | 7P0Hy00 | | Other specified diagnostic echocardiography | |  |
| Read | | 9Ee0800 | | Adult echocardiography procedure report | |  |
| Read | | R132200 | | [D]Ultrasound cardiogram abnormal | |  |
| Read | | 7P0H600 | | Contrast echocardiography | |  |
| Read | | 8A54400 | | Monitoring of cardiac output using echocardiography | |  |
| Read | | 7P0H.00 | | Diagnostic echocardiography | |  |

**B. Specialist assessment**

| **Code Type** | | **Code** | |  | | **Description** | |
| --- | --- | --- | --- | --- | --- | --- | --- |
| Read | | 8H4R.00 | | Referral to cardiology special interest general practitioner | |  |  |
| Read | | 8HVJ.00 | | Private referral to cardiologist | |  |  |
| Read | | 8H44000 | | Referral to cardiology multidisciplinary team | |  |  |
| Read | | 8H44.00 | | Cardiological referral | |  |  |
| Read | | 8HTL000 | | Referral to rapid access heart failure clinic | |  |  |
| Read | | 8HTL.00 | | Referral to heart failure clinic | |  |  |
| Read | | ZL5A100 | | Referral to cardiologist | |  |  |
| Read | | 8Hkt.00 | | Referral to community cardiology service | |  |  |

**C. Electrocardiogram**

| **Code Type** | | **Code** | |  | | **Description** |
| --- | --- | --- | --- | --- | --- | --- |
| Entity | | 217 | | Electrocardiogram | |  |
| Entity | | 304 | | ECG exercise | |  |
| Entity | | 379 | | ECG ambulatory | |  |
| Read | | R143100 | | [D]Electrocardiogram (ECG) abnormal | |  |
| Read | | 32...00 | | Electrocardiography | |  |
| Read | | 32Z..00 | | Electrocardiography NOS | |  |
| Read | | 7P0G.00 | | Diagnostic electrocardiography | |  |
| Read | | 7P0Gz00 | | Diagnostic electrocardiography NOS | |  |
| Read | | 3297.11 | | Electrocardiogram: Mobitz type 1 second degree AV block | |  |
| Read | | 329H.00 | | Electrocardiogram: Mobitz type 2 second degree AV block | |  |
| Read | | 7P0P.00 | | Other diagnostic electrocardiography | |  |
| Read | | 3216 | | ECG normal | |  |
| Read | | 32...12 | | ECG | |  |
| Read | | 3299 | | ECG: right bundle branch block | |  |
| Read | | 3214000 | | Ambulatory ECG normal | |  |
| Read | | 3217 | | ECG abnormal | |  |
| Read | | 3213000 | | Exercise ECG normal | |  |
| Read | | 3213100 | | Exercise ECG abnormal | |  |
| Read | | 3272 | | ECG: atrial fibrillation | |  |
| Read | | 3212 | | Standard ECG | |  |
| Read | | 329A.00 | | ECG: left bundle branch block | |  |
| Read | | 3282 | | ECG: ventricular tachycardia | |  |
| Read | | 326..00 | | ECG: ectopic beats | |  |
| Read | | 324..00 | | ECG:left ventricle hypertrophy | |  |
| Read | | 3273 | | ECG: atrial flutter | |  |
| Read | | 323..00 | | ECG: myocardial infarction | |  |
| Read | | 322..00 | | ECG: myocardial ischaemia | |  |
| Read | | 32L..00 | | ECG: left ventricular strain | |  |
| Read | | 329..00 | | ECG: heart block | |  |
| Read | | 321B.00 | | 12 lead ECG | |  |
| Read | | 3242 | | ECG: shows LVH | |  |
| Read | | 3262 | | ECG: extrasystole | |  |
| Read | | 327..00 | | ECG: supraventricular arrhythmia | |  |
| Read | | 3221 | | ECG: no myocardial ischaemia | |  |
| Read | | 3274 | | ECG: paroxysmal atrial tachy. | |  |
| Read | | 3297 | | ECG: Wenckebach phenomenon | |  |
| Read | | 328..00 | | ECG: ventricular arrhythmia | |  |
| Read | | 321..00 | | ECG - general | |  |
| Read | | 3263 | | ECG: ventricular ectopics | |  |
| Read | | 324Z.00 | | ECG: LVH NOS | |  |
| Read | | 32F2.00 | | ECG: T wave abnormal | |  |
| Read | | 32E3.00 | | ECG: S-T elevation | |  |
| Read | | 32F4.00 | | ECG: T wave inverted | |  |
| Read | | 32FZ.00 | | ECG: T wave NOS | |  |
| Read | | 32J2.00 | | ECG: QRS complex abnormal | |  |
| Read | | 3294 | | ECG:partial A-V block-long P-R | |  |
| Read | | 3234 | | ECG:posterior/inferior infarct | |  |
| Read | | 3222 | | ECG:shows myocardial ischaemia | |  |
| Read | | 3231 | | ECG: no myocardial infarction | |  |
| Read | | 3233 | | ECG: antero-septal infarct. | |  |
| Read | | 329Z.00 | | ECG: heart block NOS | |  |
| Read | | 3241 | | ECG: no LVH | |  |
| Read | | 328Z.00 | | ECG: ventricular arrhythmia NOS | |  |
| Read | | 3283 | | ECG: ventricular fibrillation | |  |
| Read | | 3291 | | ECG: no heart block | |  |
| Read | | 32B..00 | | ECG: Q wave | |  |
| Read | | 322Z.00 | | ECG: myocardial ischaemia NOS | |  |
| Read | | 3293 | | ECG:complete sinu-atrial block | |  |
| Read | | 325..00 | | ECG:right ventricle hypertrop. | |  |
| Read | | 3232 | | ECG: old myocardial infarction | |  |
| Read | | 3295 | | ECG: partial A-V block - 2:1 | |  |
| Read | | 3298 | | ECG: complete A-V block | |  |
| Read | | 32E4.00 | | ECG: S-T depression | |  |
| Read | | 32A3.00 | | ECG: P mitrale | |  |
| Read | | 32C2.00 | | ECG: R wave abnormal | |  |
| Read | | 326Z.00 | | ECG: ectopic beats NOS | |  |
| Read | | 32K3.00 | | ECG: Q-T interval prolonged | |  |
| Read | | 32E2.00 | | ECG: S-T interval abnormal | |  |
| Read | | 32B2.00 | | ECG: Q wave abnormal | |  |
| Read | | 32EZ.00 | | ECG: S-T interval NOS | |  |
| Read | | 32D2.00 | | ECG: S wave abnormal | |  |
| Read | | 32F3.00 | | ECG: T wave flattened | |  |
| Read | | 32I3.00 | | ECG: P-R interval prolonged | |  |
| Read | | 32E1.00 | | ECG: S-T interval normal | |  |
| Read | | 327Z.00 | | ECG: supraventric. arryth. NOS | |  |
| Read | | 3236 | | ECG: lateral infarction | |  |
| Read | | 3292 | | ECG: partial sinu-atrial block | |  |
| Read | | 321C.00 | | ECG sinus rhythm | |  |
| Read | | 32A2.00 | | ECG: P wave abnormal | |  |
| Read | | 3235 | | ECG: subendocardial infarct | |  |
| Read | | 32F..00 | | ECG: T wave | |  |
| Read | | 3271 | | ECG: no supraventric. arryth. | |  |
| Read | | 32E..00 | | ECG: S-T interval | |  |
| Read | | 32I2.00 | | ECG: P-R interval abnormal | |  |
| Read | | 32A..00 | | ECG: P wave | |  |
| Read | | 323Z.00 | | ECG: myocardial infarct NOS | |  |
| Read | | 32J1.00 | | ECG: QRS complex normal | |  |
| Read | | 3252 | | ECG: shows RVH | |  |
| Read | | 3251 | | ECG: no RVH | |  |
| Read | | 32C..00 | | ECG: R wave | |  |
| Read | | 325Z.00 | | ECG: RVH NOS | |  |
| Read | | 32A4.00 | | ECG: P pulmonale | |  |
| Read | | 32B3.00 | | ECG: Q wave pathological | |  |
| Read | | 32AZ.00 | | ECG: P wave NOS | |  |
| Read | | 32B1.00 | | ECG: Q wave normal | |  |
| Read | | 32K..00 | | ECG: Q-T interval | |  |
| Read | | 32CZ.00 | | ECG: R wave NOS | |  |
| Read | | 32J3.00 | | ECG: QRS complex prolonged | |  |
| Read | | 32I4.00 | | ECG: P-R interval shortened | |  |
| Read | | 32C1.00 | | ECG: R wave normal | |  |
| Read | | 32I..00 | | ECG: P-R interval | |  |
| Read | | 32BZ.00 | | ECG: Q wave NOS | |  |
| Read | | 32G..00 | | ECG: U wave | |  |
| Read | | 32K1.00 | | ECG: Q-T interval normal | |  |
| Read | | 32C3.00 | | ECG: R wave tall | |  |
| Read | | 32IZ.00 | | ECG: P-R interval NOS | |  |
| Read | | 32K2.00 | | ECG: Q-T interval abnormal | |  |
| Read | | 32G1.00 | | ECG: U wave normal | |  |
| Read | | 32G2.00 | | ECG: U wave abnormal | |  |
| Read | | 32K4.00 | | ECG: Q-T interval shortened | |  |
| Read | | 32D3.00 | | ECG: S wave deep | |  |
| Read | | 32F1.00 | | ECG: T wave normal | |  |
| Read | | 32KZ.00 | | ECG: Q-T interval NOS | |  |
| Read | | 3261 | | ECG: no ectopic beats | |  |
| Read | | 32A1.00 | | ECG: P wave normal | |  |
| Read | | 32JZ.00 | | ECG: QRS complex NOS | |  |
| Read | | 3281 | | ECG: no ventricular arrhythmia | |  |
| Read | | 32I1.00 | | ECG: P-R interval normal | |  |
| Read | | 32D1.00 | | ECG: S wave normal | |  |
| Read | | 3296 | | ECG: partial A-V block - 3:1 | |  |
| Read | | 32J..00 | | ECG: QRS complex | |  |
| Read | | 329B.00 | | ECG: trifascicular block | |  |
| Read | | 329D.00 | | ECG: left anterior fascicular block | |  |
| Read | | 329C.00 | | ECG: bifascicular block | |  |
| Read | | 329F.00 | | ECG: right bundle branch and left anterior fascicular block | |  |
| Read | | 32DZ.00 | | ECG: S wave NOS | |  |
| Read | | 329G.00 | | ECG: right bundle branch and left posterior fascicular block | |  |
| Read | | 329E.00 | | ECG: left posterior fascicular block | |  |

**D. Natriuretic peptides**

| **Code Type** | **Code** |  | **Description** |
| --- | --- | --- | --- |
| Read | 44AR.00 |  | Plasma B natriuretic peptide level |
| Read | 44AF.00 |  | Brain natriuretic peptide level |
| Read | 44AN.00 |  | Plasma pro-brain natriuretic peptide level |
| Read | 44AP.00 |  | Serum pro-brain natriuretic peptide level |
