# Supplementary material for: Diagnostic tests, drug prescriptions, and follow-up patterns after incident heart failure: A cohort study of 93,000 UK patients
Source: PLoS Med. 2019 May 21;16(5):e1002805. doi: 10.1371/journal.pmed.1002805 (PMC6528949; doi:10.1371/journal.pmed.1002805)
Supplement: S5 Table — ESC, European Society of Cardiology; NICE, National Institute for Clinical Excellence. (DOCX) [file pmed.1002805.s010.docx]

S5 Table: Recommended maintenance dosages of pharmacological treatments indicated in patients with heart failure and reduced ejection fraction, as per ESC and NICE guidelines during the study period.

| Drug Class | Drug Name | Daily Target Dose | | | | | |  | | | |
| --- | --- | --- | --- | --- | --- | --- | --- | --- | --- | --- | --- |
|  |  | Unique value defined for the purpose of this study | European Society of Cardiology guidelines | | | | NICE guidelines | | | |  |
|  |  |  | **2001** | **2005** | **2008** | **2012** | | | **2003** |  |  |
| ACE inhibitors | Captopril | 150 mg | 75-150 mg | 75-150 mg | 150-300 mg | 150 mg | | | 150-300 mg |  |  |
| ACE inhibitors | Cilazapril | 1 mg | 1-2.5 mg | - | - | - | | | 1-2.5 mg |  |  |
| ACE inhibitors | Enalapril | 20 mg | 20 mg | 20 mg | 20-40 mg | 20-40 mg | | | 20-40 mg |  |  |
| ACE inhibitors | Fosinopril | 20 mg | 20 mg | - | - | - | | | 40 mg |  |  |
| ACE inhibitors | Lisinopril | 20 mg | 5-20 mg | 5-20 mg | 20-35 mg | 20-35 mg | | | 30-35 mg |  |  |
| ACE inhibitors | Perindopril | 4 mg | 4 mg | - | - |  | | | 4 mg |  |  |
| ACE inhibitors | Quinapril | 5 mg | 5-10mg | - | - |  | | | 10-20 mg |  |  |
| ACE inhibitors | Ramipril | 10 mg | 5-10 mg | 5-10 mg | 10 mg | 10 mg | | | 10 mg |  |  |
| ACE inhibitors | Trandolapril | 4 mg | 4 mg | 4 mg | 4 mg | 4 mg | | | - |  |  |
| ARB | Candesartan | 32 mg | 4-16 mg | 4-32 mg | 32 mg | 32 mg | | | 4-16 mg |  |  |
| ARB | Losartan | 150 mg | 50-100 mg | 50-100 mg | - | 150 mg | | | 50-100 mg |  |  |
| ARB | Valsartan | 320 mg | 80-320 mg | 80-320 mg | 320 mg | 320 mg | | | 80-320 mg |  |  |
| Beta Blocker | Bisoprolol | 10 mg | 10 mg | 10 mg | 10 mg | 10 mg | | | 10 mg |  |  |
| Beta Blocker | Carvedilol | 50 mg | 50 mg | 50 mg | 50-100 mg | 50-100 mg | | | 50-100 mg |  |  |
| Beta Blocker | Metoprolol succinate | 200 mg | 200 mg | 200 mg | 200 mg | 200 mg | | | - |  |  |
| Beta Blocker | Metoprolol tartrate | 150 mg | 150 mg | - | - | - | | | - |  |  |
| Beta Blocker | Nebivolol | 10 mg | - | 10 mg | 10 mg | 10 mg | | | - |  |  |
| MRA | Eplerenone | 50 mg | - | 25 mg | 50 mg | 50 mg | | | - |  |  |
| MRA | Spironolactone | 25 mg | 25 mg | 12.5-25 mg | 25-50 mg | 25-50 mg | | | 12.5-25 mg |  |  |

***Notes****: The evidence-base underlying heart failure treatment recommendations has changed over the study period (2002-2014). So as to compare changes in drug doses over the time, we included all drugs referred to in the guidelines applicable during the study period, and defined a unique target dose for the purpose of this study as the minimal recommended target dose in the latest guideline available during the study period. We further highlight in blue those drugs that are considered evidence-based treatments as per 2016 ESC guidelines. The 2010 NICE guidelines do not contain target dosages for the treatment of heart failure.*

***Abbreviations****: ACE = angiotensin-converting enzyme; ARB = angiotensin receptor blocker; MRA = mineralocorticoid receptor antagonist; NICE =* National Institute for Health and Clinical Excellence.
