# Supplementary material for: Diagnostic tests, drug prescriptions, and follow-up patterns after incident heart failure: A cohort study of 93,000 UK patients
Source: PLoS Med. 2019 May 21;16(5):e1002805. doi: 10.1371/journal.pmed.1002805 (PMC6528949; doi:10.1371/journal.pmed.1002805)
Supplement: S6 Table — (DOCX) [file pmed.1002805.s011.docx]

S6 Table: Baseline characteristics of patients with incident heart failure, by record of ejection fraction.

| **Characteristic** | **All patients** (n=93,074) | **Ejection fraction record** | |
| --- | --- | --- | --- |
|  |  | **Reduced  ejection fraction**  (n= 11,040, 12%) | **Preserved or unspecified  ejection fraction**  (n= 82,034, 88%) |
| Age [years], mean (SD) | 76.7 (12.6) | 69.2 (13.7) | 77.8 (12) |
| Women, no. (%) | 45,647 (49%) | 3,853 (35%) | 41,794 (51%) |
| Ethnicity, no. (%) |  |  |  |
| White | 56,011 (88%) | 7,067 (86%) | 48,944 (88%) |
| *Missing* | *29,122 (31%)* | 2,819 (26%) | 26,303 (32%) |
| Socioeconomic status, no. (%) |  |  |  |
| 1 (least deprived) | 18,371 (20%) | 2,366 (21%) | 16,005 (20%) |
| 2 | 20,073 (22%) | 2,450 (22%) | 17,623 (21%) |
| 3 | 20,052 (22%) | 2,360 (21%) | 17,692 (22%) |
| 4 | 18,308 (20%) | 2,061 (19%) | 16,247 (20%) |
| 5 (most deprived) | 16,270 (17%) | 1,803 (16%) | 14,467 (18%) |
| Systolic blood pressure |  |  |  |
| Mean (SD) [mmHg] | 133 (21) | 129.6 (19.5) | 133.1 (20.6) |
| *Missing, no. (%)* | *5,195 (6%)* | 390 (3%) | 4,805 (6%) |
| Diastolic blood pressure |  |  |  |
| Mean (SD) [mmHg] | 74 (12) | 75.1 (11.2) | 74.4 (11.5) |
| *Missing, no. (%)* | *5,195 (6%)* | 403 (3%) | 4,792 (6%) |
| BMI category, no. (%) |  |  |  |
| Underweight | 2,193 (4%) | 151 (2%) | 2,042 (4%) |
| Normal | 17,381 (31%) | 2,117 (28%) | 15,264 (32%) |
| Overweight | 18,786 (34%) | 2,835 (37%) | 15,951 (33%) |
| Obese | 17,644 (31%) | 2,524 (33%) | 15,120 (31%) |
| *Missing* | *37,070 (40%)* | 3,413 (31%) | 33,657 (41%) |
| Smoking, no. (%) |  |  |  |
| No | 29,551 (41%) | 3,575 (38%) | 25,976 (42%) |
| Ex | 32,572 (45%) | 4,520 (48%) | 28,052 (45%) |
| Yes | 9,596 (13%) | 1,382 (15%) | 8,214 (13%) |
| *Missing* | *21,355 (23%)* | 1,563 (14%) | 19,792 (24%) |
| Comorbidities |  |  |  |
| Atrial fibrillation, no. (%) | 36,950 (40%) | 3,681 (33%) | 33,269 (41%) |
| Chronic kidney disease, no. (%) | 22,762 (24%) | 1,961 (18%) | 20,801 (25%) |
| Chronic obstructive pulmonary  disease, no. (%) | 17,896 (19%) | 1,456 (13%) | 16,440 (20%) |
| Diabetes, no. (%) | 20,531 (22%) | 2,080 (19%) | 18,451 (22%) |
| Dyslipidaemia, no. (%) | 25,958 (28%) | 3,420 (31%) | 22,538 (27%) |
| Hypertension, no. (%) | 62,419 (67%) | 6,464 (59%) | 55,955 (68%) |
| Ischaemic heart disease, no. (%) | 45,584 (49%) | 5,420 (49%) | 40,164 (49%) |
| Osteoarthritis, no. (%) | 40,176 (43%) | 4,012 (36%) | 36,164 (44%) |
| 3 or more comorbidities, no. (%) | 73,610 (79%) | 7,555 (68%) | 66,055 (81%) |

*Ejection fraction classification as recorded in patients’ primary care record. Number and percentage of records with missing data are displayed for variables with missing entries. Category percentages refer to complete cases. Socioeconomic status refers to Index of Multiple Deprivation (IMD) 2015 quintile, with 1 referring to the most affluent and 5 to the most deprived quintile. Number of comorbidities refers to any of the 17 conditions investigated (see Methods).* ***Abbreviations****: HF = heart failure.*
