# Supplementary material for: Diagnostic tests, drug prescriptions, and follow-up patterns after incident heart failure: A cohort study of 93,000 UK patients
Source: PLoS Med. 2019 May 21;16(5):e1002805. doi: 10.1371/journal.pmed.1002805 (PMC6528949; doi:10.1371/journal.pmed.1002805)
Supplement: S7 Table — (DOCX) [file pmed.1002805.s012.docx]

S7 Table: Temporal trends in supplementary care indicators following incident heart failure, by year of diagnosis.

|  | **Denominator cohort (n)** | **2002-2014** | **2002** | **2014** |  |
| --- | --- | --- | --- | --- | --- |
| **Treatment dose, among patients initiated on therapy** | Patients diagnosed or followed-up in primary care, reduced ejection fraction (11,042) and no drug-specific contra-indication, and initiated on therapy within 3 months. | **mean (SD)** | **mean (SD)** | **mean (SD)** | **Adjusted difference [95% CI]** |
| ACE-I / ARB | 7,750 | 60% (43%) | 64% (42%) | 51% (40%) | -0.12 [-0.18, -0.06] |
| Beta-blocker | 4,663 | 42% (30%) | 39% (31%) | 39% (32%) | 0.03 [-0.05, 0.1] |
| MRA | 1,999 | 83% (49%) | 86% (45%) | 66% (41%) | -0.17 [-0.31, -0.03] |
| Beta-blocker and ACE-I / ARB | 3,759 | 52% (29%) | 54% (26%) | 46% (28%) | -0.07 [-0.15, 0.01] |
| **Treatment uptitration, up to 50% of target dose** | Patients diagnosed or followed-up in primary care, reduced ejection fraction (11,042) and no drug-specific contra-indication | **mean (SD)** | **mean (SD)** | **mean (SD)** | **Risk ratio [95% CI]** |
| ACE-I / ARB | 9,922 | 5,716 (58%) | 240 (63%) | 367 (50%) | 0.80 [0.72, 0.89] |
| Beta-blocker | 8,432 | 2,490 (30%) | 41 (13%) | 224 (33%) | 2.73 [2.06, 3.61] |
| MRA | 10,898 | 2,784 (26%) | 93 (23%) | 278 (35%) | 1.58 [1.25, 2.01] |
| Beta-blocker and ACE-I / ARB | 7,770 | 1,671 (22%) | 29 (10%) | 143 (22%) | 2.51 [1.68, 3.75] |

*Risk* *ratios or adjusted differences, and 95% confidence intervals (CI) compare 2014 to 2002, adjusting for year of diagnosis, age, sex, socioeconomic status and region. Treatment dose presents the average daily dose prescribed in the first 12 months following incident heart failure, as % of guideline-recommended target dose. Treatment uptitration refers to the issue of at least 1 prescription at 50% of the recommended target dose in the 12 months following diagnosis.* ***Abbreviations****: HF = heart failure, ACE-I = angiotensin-converting-enzyme inhibitor, ARB = angiotensin receptor blocker, MRA =* mineralocorticoid receptor antagonists.
