# Supplementary material for: Diagnostic tests, drug prescriptions, and follow-up patterns after incident heart failure: A cohort study of 93,000 UK patients
Source: PLoS Med. 2019 May 21;16(5):e1002805. doi: 10.1371/journal.pmed.1002805 (PMC6528949; doi:10.1371/journal.pmed.1002805)
Supplement: S8 Table — (DOCX) [file pmed.1002805.s013.docx]

S8 Table: Diagnostic investigations following incident heart failure, stratified by age and sex.

| **Age group** (years) | **Full cohort**  **(n = 47,925)** | **Men**  **(n=25,848)** | **Women**  **(n=22,077)** | **Risk ratio  [95% CI]** |
| --- | --- | --- | --- | --- |
| <45 | 750 (66%) | 516 (70%) | 234 (58%) | 0.84 [0.76, 0.92] |
| 45-54 | 1,684 (76%) | 1,248 (78%) | 436 (73%) | 0.93 [0.88, 0.98] |
| 55-64 | 4,092 (78%) | 2,875 (79%) | 1,217 (77%) | 0.97 [0.94, 1.00] |
| 65-74 | 7,890 (76%) | 4,984 (77%) | 2,906 (74%) | 0.97 [0.95, 0.99] |
| 75+ | 19,244 (66%) | 9,504 (71%) | 9,740 (63%) | 0.90 [0.89, 0.92] |

*Risk ratios and 95% confidence intervals (CI) compare women to men, adjusting for year of diagnosis, sex, socioeconomic status and region. Diagnostic investigations refer to any of echocardiogram, electrocardiogram, natriuretic peptide test, or specialist assessment referred for within ± 6 months of incident heart failure.*
