# Supplementary material for: Diagnostic tests, drug prescriptions, and follow-up patterns after incident heart failure: A cohort study of 93,000 UK patients
Source: PLoS Med. 2019 May 21;16(5):e1002805. doi: 10.1371/journal.pmed.1002805 (PMC6528949; doi:10.1371/journal.pmed.1002805)
Supplement: S1 Fig — (DOCX) [file pmed.1002805.s014.docx]

Figure S1: Average daily dose of guideline-recommended treatments prescribed around the time of incident heart failure, by time period of diagnosis.

*Average daily dose prescribed to patients with heart failure and reduced ejection fraction without drug-specific contraindications or intolerances, from 3 months prior up to 12 months following incident heart failure, in patients diagnosed between 2012 and 2014 and in those diagnosed between 2002 and 2004. Average daily dose is expressed as a percentage of the guideline-recommended target dose.* ***Abbreviations****: ACE-I = angiotensin-converting-enzyme inhibitor, ARB = angiotensin receptor blocker, MRA =* mineralocorticoid receptor antagonists.
